# Transcriptomic Landscape and Regulatory Pathways of Drought Response in Rice (*Oryza sativa* L.): A Meta-Analysis of Microarray and RNA-Seq Data

**DOI:** 10.3390/ijms27073167

**Published:** 2026-03-31

**Authors:** Maria Kampa, Konstantinos Makropoulos, Aikaterini Goule, Ioannis A. Tamposis, Panagiota I. Kontou, Pantelis G. Bagos, Georgia G. Braliou

**Affiliations:** 1 Department of Computer Science and Biomedical Informatics, University of Thessaly, 35131 Lamia, Greece; mkampa@uth.gr (M.K.); makkonstant@uth.gr (K.M.); itamposis@uth.gr (I.A.T.); pbagos@compgen.org (P.G.B.); 2 Department of Mathematics, University of Thessaly, 35132 Lamia, Greece; pkontou@uth.gr

**Keywords:** *Oryza sativa*, NGS, RNA-Seq, microarray, meta-analysis, drought stress, differentially expressed genes, protein–protein interaction network, regulatory pathways, enriched pathways

## Abstract

Drought significantly disrupts rice productivity under increasing climate volatility. Identifying robust molecular determinants for resilience remains a critical priority for crop improvement. Following the PRISMA guidelines, we performed a large-scale, dual-platform meta-analysis of RNA-Seq and microarray datasets to elucidate the robust transcriptomic landscape of *Oryza sativa* underwater deficit. Tissue-specific regulatory pathways were identified using STRING, g:Profiler, and PANTHER. Our analysis resolved distinct functional divergence, where shoots prioritize photosynthetic adjustment while roots emphasize transcriptional and chromatin reprogramming. Beyond validating core ABA signaling, we uncover a novel metabolic pivot: the activation of glyoxylate and dicarboxylate metabolism to mitigate drought-induced carbon starvation. We further identify specialized transport systems for ions and electrons across organelle membranes, alongside cellular reorganization driven by autophagy and actin-dependent cytoskeleton remodeling. These findings highlight a sophisticated network of survival strategies governing energy conservation and structural adaptation. By synthesizing heterogeneous transcriptomics, this study reveals robust pathways that are overlooked in single-platform investigations. This work provides a prioritized roadmap for utilizing functional validation and precision breeding to accelerate the development of climate-resilient rice cultivars.

## 1. Introduction

Rice (*Oryza sativa* L.) is a major staple crop and a primary source of calories for more than half of the world’s population, playing a central role in global food security alongside wheat and maize [1]. The global rice production reached nearly 790 million metric tons in 2021, and the demand is expected to increase further with continued population growth, underscoring the importance of improving rice productivity under future food security challenges [2,3]. However, the sustainability of this crucial crop is increasingly threatened by climatic challenges that lead to various environmental stressors. Over the past decade, global agriculture has been severely affected by rising temperatures and unpredictable weather patterns [4]. Among the various abiotic stressors, drought stands out as one of the most critical constraints on rice productivity, particularly where rice is a dietary staple for billions of people, thereby endangering food security in vulnerable regions, such as Asia [5,6,7]. Drought stress disrupts critical physiological and developmental processes in rice [8,9], posing a worldwide necessity and challenge to further understand the molecular mechanisms that govern drought resistance [10].

The emerging genomic and biotechnological approaches [11], together with recent advancements in high-throughput technologies, such as microarray analyses and RNA sequencing (RNA-seq), have revolutionized the field of plant transcriptomics by enabling the simultaneous examination of thousands of genes under specific environmental conditions [12,13,14,15]. These approaches provide insights and help identify the molecular pathways governing stress responses and developmental processes. Such knowledge is critical for guiding the development of improved rice varieties with enhanced adaptability to challenging and changing environments [16,17].

To maximize the informative value of these large-scale datasets, meta-analysis has emerged as a powerful statistical framework. By synthesizing transcriptomics data across multiple studies, meta-analysis substantially increases the statistical power, reduces bias, and reveals genes and pathways that may have remained undetected in individual investigations [18,19,20,21,22,23]. When applied to RNA-seq and microarray data across diverse conditions and genotypes, meta-analysis can uncover robust gene expression signatures and key genetic determinants of complex traits such as drought resilience. Thus, recruiting meta-analysis into plant transcriptomics offers a rigorous route to novel biological insights and accelerates the discovery of resilience-enhancing gene signatures.

In this study, we conducted meta-analyses of publicly available RNA-seq and microarray datasets to evaluate the transcriptomic responses of *Oryza sativa* under drought stress. We focused on two tissues that are central to drought adaptation: seedling/shoot tissues, which are pivotal for development, photosynthetic assimilation, and stress signaling, and roots, which govern nutrient–water uptake and hydro-osmotic regulation. Our study not only contributes to a better understanding of key molecular pathways but also provides novel drought-responsive genes that underpin drought resilience. This study provides a significant contribution to the field, offering data that can be used in conjunction with genotype-based meta-analyses to accelerate research into emerging climate threats. This study may aid in accelerating the development of climate-resilient rice cultivars, ensuring sustainable productivity under shifting environmental conditions.

## 2. Results

**Figure 1:**
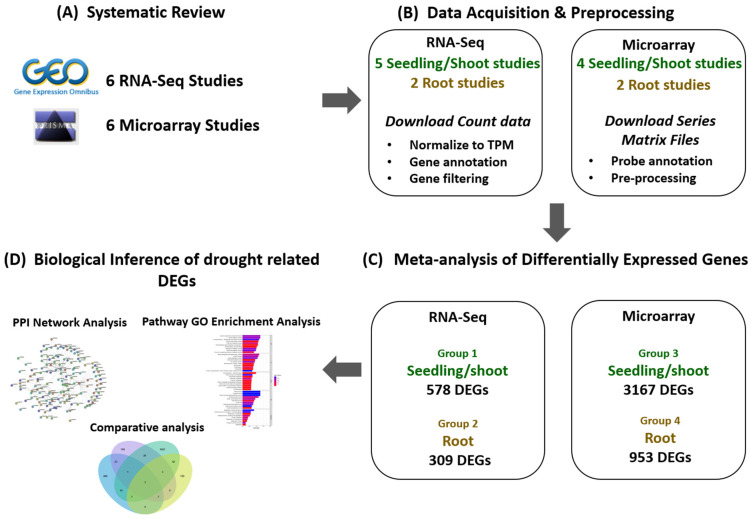
The workflow diagram of the methodology employed in this study.

In this study, we integrated large-scale transcriptomic datasets to robustly identify and characterize genes that are differentially expressed in rice under normal versus water-deficient conditions. Because seedlings are typically categorized as shoots, we analyzed two tissue classes: seedling/shoot and root. The overall workflow is outlined in Figure 1. The thorough review of the literature yielded four dataset groups: Group 1: seedling/shoot, RNA-seq; Group 2: root, RNA-seq; Group 3: seedling/shoot, microarray; and Group 4: root, microarray.

### 2.1. Study Characteristics

**Figure 2:**
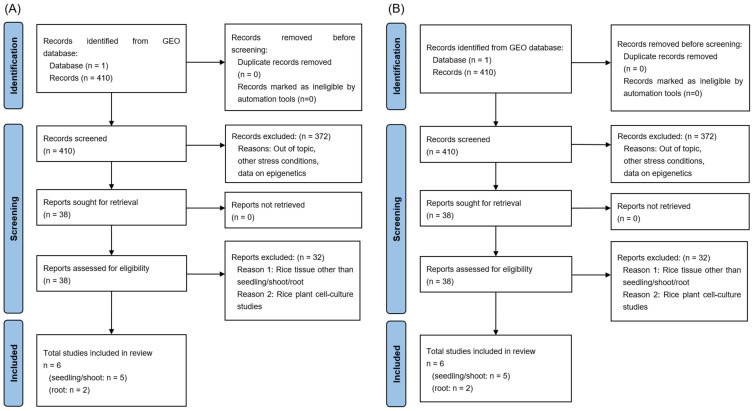
The PRISMA flow diagrams to retrieve studies with data for seedling/shoot or root tissues obtained from RNA-seq (**A**) and microarray (**B**) methodologies.

A total of 410 RNA-Seq and 592 microarray studies were retrieved from the GEO database and screened in accordance with the PRISMA guidelines (Figure 2A and Figure 2B, respectively). From the RNA-Seq data acquisition (Figure 1A), three studies were finally considered eligible for seedling, two for shoot (five in total for seedling/shoot), and two for rice root tissue. The detailed characteristics of the included RNA-Seq studies are provided in Table 1. Concerning the microarray data acquisition, three studies for seedling, one for shoot (four in total for seedling/shoot), and two studies for rice root tissue were considered eligible. The detailed characteristics of the included microarray studies are presented in Table 2. 

### 2.2. Differentially Expressed Genes (DEGs) from RNA-Seq and Microarray Data Obtained Through Meta-Analysis

A meta-analysis was conducted with data from each of the four distinct dataset groups as illustrated in Figure 1C. To minimize false positives DEGs, a sensitivity analysis was conducted with multiple comparison tests across progressively stringent FDR thresholds. The results from the meta-analysis of the RNA-Seq data (Groups 1 and 2) for multiple FDR, along with the numbers of retrieved DEGs, are shown in Table 3, while the respective results obtained from microarray studies (Groups 3 and 4) are shown in Table 4.

To ensure the robustness of the outcome and minimize false positives, we implemented a stringent statistical threshold of an FDR < 0.001 for the identification of differentially expressed genes (DEGs). Consequently, the meta-analysis with an FDR < 0.001 revealed 578 DEGs for seedling/shoots RNA-Seq (Group 1) and 309 DEGs for root RNA-Seq (Group 2) (Table 3 and Supplementary File S1a,b). Similarly, the meta-analysis with the microarray data revealed 3167 DEGs for seedling/shoots (Group 3, Supplementary File S1c) and 953 DEGs for roots at the same FDR < 0.001 significance level (Group 4, Supplementary File S1d).

To quantify the added value of the present meta-analysis, we calculated the integration-driven discovery rate (IDR) and the integration-driven revision rate (IRR) [36,37]. The high IRR values (ranging from 86.4% to 97.7%) across all groups (Table 5 and Supplementary File S8) demonstrate the ability of the meta-analysis to filter out a significant proportion of potentially false-positive or study-specific ‘noise’ that appears in single studies due to limited sample sizes. Crucially, the meta-analysis identified a significant percentage of DEGs (IDR) that were not detected in any individual study. The IDR highlights the discovery of genes that lacked statistical significance in individual studies but reached the significance threshold when pooled [38]. For instance, in the microarray root analysis, 78.7% of the identified DEGs were novel discoveries made possible only by the increased statistical power of our integrated approach (Table 5). These results underscore the critical role of meta-analysis in uncovering stress signatures in tissues where individual datasets may be underpowered.

To visually assess the degree of consensus between the meta-analysis and the individual experiments, we performed a comparative intersection analysis (Supplementary Figure S3). The Venn diagrams reveal a notable lack of overlap between the DEGs reported in the original studies and those identified through our meta-analytical framework. In the seedling/shoot RNA-Seq datasets (Supplementary Figure S3A,B), individual studies frequently reported exhaustive lists of thousands of DEGs (e.g., 13,875 in GSE160679); however, our meta-analysis prioritized a much more refined set of 578 high-confidence genes. This corrective power was even more evident in the microarray seedling/shoot data, where the meta-approach performed a massive revision, narrowing nearly 18,000 DEGs from a single study (GSE115826) down to a robust core of 3167 genes. For root tissues, the meta-analysis proved essential for discovering ‘hidden’ information. In the root RNA-Seq contrast (Supplementary Figure S3C), the intersection with the original reports was remarkably low—sharing only nine genes with GSE147158—proving that our method captures biological signals that were previously overlooked. Similarly, in root microarray datasets (Supplementary Figure S3G,H), the meta-analysis successfully identified 953 consistent DEGs, even where individual studies showed little consensus. These comparisons highlight the two primary advantages of this approach: it effectively filters out study-specific noise (reflected in our high IRR values) while extracting a robust, core drought-responsive signature that remains undetected in single-study analyses.

### 2.3. Protein–Protein Interaction Networks and Protein Clusters of DEGs from RNA-Seq and Microarray Meta-Analysis

To elucidate the functional coordination of drought-responsive DEGs in rice tissues, we analyzed their protein–protein interaction (PPI) network using the STRING database. This approach enables the identification of central regulatory hubs and potential stress-associated pathways that are implicated in drought adaptation and resilience (Figure 1D).

**Figure 3:**
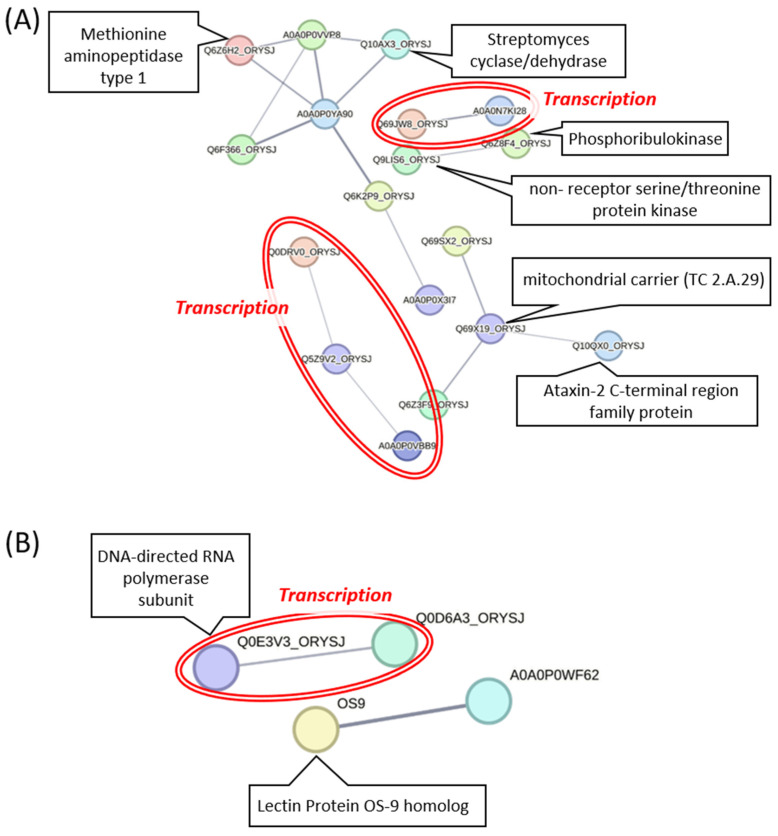
Protein–protein interaction networks from STRING for the DEGs that were identified through the meta-analysis of RNA-Seq data from (**A**) seedling/shoot (Group 1) and (**B**) root tissues (Group 2). The edges indicate experimentally determined interactions. The double-lined red ellipses denote common functional groups between seedling/shoot (**A**) and root tissues (**B**).

To this end, a PPI network was constructed for the 578 DEGs that were revealed from the seedling/shoot RNA-Seq data (Group 1) meta-analysis (Figure 3A; Supplementary File S1a). Of the 578 DEGs, only 460 were recognized by STRING, and only 18 genes displayed strong interconnections (only experimentally verified interactions, with a confidence interaction cutoff score of 0.4 and an interaction strength ≥ 0.01). Of these 18 rice genes (Supplementary File S2a), six were assigned descriptive functional names based on the biological information retrieved from the STRING database and are shown in Figure 3A. Since the PPI network did not show significant enrichment—indicating limited known interactions among the identified DEGs—we further assessed their biological relevance through a domain analysis in SMART, which is a database linked to functional annotation in STRING db. This additional step provided insights into potential roles or processes that were not captured by the biological information protein databases. Through this analysis, seven of the interconnected nodes [A0A0P0YA90 (Os12g0488600), A0A0P0VVP8 (Os03g0259500), Q10AX3_ORYSJ (Os03g0837900), Q6Z6H2_ORYSJ (Os02g0761700-methionine aminopeptidase type 1), Q6F366_ORYSJ (Os05g0575300), Q6K2P9_ORYSJ (Os09g0326900), and A0A0P0X3I7 (Os07g0180800)] were associated with ribosomal functions, suggesting a potential role in maintaining or adjusting protein synthesis under drought stress. Five genes [Os06g0267500 (Q5Z9V2_ORYSJ), Os01g0881000 (A0A0P0VBB9), Os03g0352500 (Q0DRV0_ORYSJ), Os02g0575700 (Q69JW8_ORYSJ), and Os03g0756500 (A0A0N7KI28)] were linked to transcription, encountering genes that are involved in chromatin remodeling complexes, transcription factors, or RNA-associated proteins, indicating possible involvement in genomic stability, repair mechanisms, or transcriptional regulation during stress. Four nodes [Q69SX2_ORYSJ (Os06g0360300), Q69X19_ORYSJ (Os06g0604500 mitochondrial substrate carrier TC 2.A.29), Q6K2G6_ORYSJ (Os08g0553800), and Q10QX0_ORYSJ (Os03g0180300, Ataxin-2 C-terminal region)] were identified as part of mitochondrial carrier systems, possibly reflecting changes in energy transport and cellular metabolism of seedling/shoot tissue to survive drought stress (Supplementary File S2a).

The PPI network of the root DEGs identified through the RNA-Seq data (Group 2) meta-analysis was assessed using STRING to explore potential functional interactions among drought-responsive genes in root tissue (Figure 3B; Supplementary File S1b). Of 309 DEGs, 281 were recognized by STRING for network analysis using experimentally validated interactions (strength interaction ≥0.01, interaction score 0.4). The network contained only four connected nodes (Figure 3B). Due to limited connectivity and lack of network enrichment, functional annotation in STRING through domain analysis in SMART was performed (Supplementary File S2b). Two gene products, Os02g0152800 (Q0E3V3_ORYSJ) and Os07g0497100 (Q0D6A3_ORYSJ), were linked to transcriptional regulation (RNA polymerase II activity and chromatin organization, respectively). Os06g0644800 (OS9) and Os04g0624900 (A0A0P0WF62) were associated with ubiquitin-dependent protein degradation, suggesting a possible role in protein quality control under stress (Supplementary File S2b). In summary, by applying a consistent functional classification across both tissues using the RNA-Seq data, transcriptional regulation emerged as a primary shared functional category, highlighting its conserved role in drought response of both seedling/shoot and root tissues.

**Figure 4:**
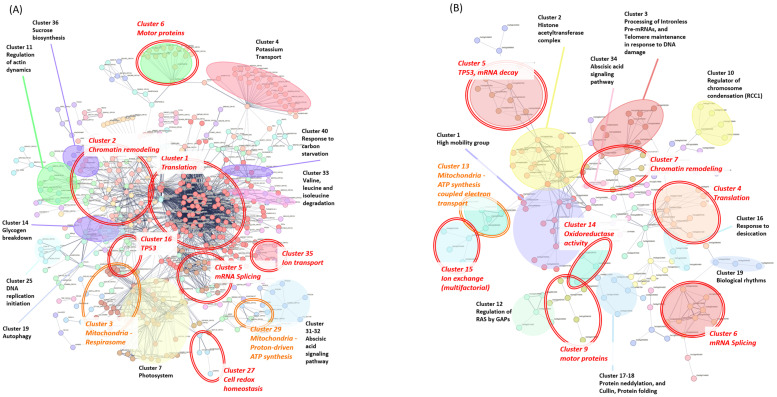
The STRING protein–protein interaction networks corresponding to: (**A**) the seedling/shoot tissue DEGs from the microarray meta-analysis (Group 3) and (**B**) the root tissue DEGs from the microarray meta-analysis (Group 4). The clusters representing groups of highly connected nodes are marked with ellipses and corresponding descriptions. The edges between the nodes indicate experimentally determined interactions. The double-lined red ellipses denote common functional groups between seedling/shoot (**A**) and root tissues (**B**), while the orange ellipses denote related biological processes.

Similarly, potential interactions among the 3167 DEGs identified from the microarray meta-analysis of seedling/shoot (Group 3) tissues were investigated (Figure 4A). Since the STRING online tool can process a maximum of 2000 proteins per analysis, the 3167 DEGs from the Group 3 meta-analysis (Supplementary File S1c) were divided into two batches. STRING recognized 2133 protein names in total, from which the top 2000, ranked by the smallest *p*-values, were selected for analysis. The resulting PPI network consisted of 524 nodes (Figure 4A and Supplementary File S3a) and 2565 edges, based on experimentally validated interactions, strength interaction of ≥0.01, and interaction score of 0.4 (Figure 4A). The STRING network was clustered using the Markov Cluster Algorithm (MCL) with an inflation parameter of two and resulted in 60 distinct clusters (Supplementary File S3a and Figure 4A). Some of these DEGs are involved in hormonal and stress-signaling regulation. Particularly, Clusters 31 and 32, which were enriched for ABA-responsive genes, highlight the activation of the abscisic acid signaling pathway, which is a central regulator of drought, osmotic stress, and stomatal adaptation, while Cluster 46, which is associated with steroid biosynthesis, suggests a complementary modulation of hormone-mediated growth and protective responses. The DEGs from Cluster 7 are photosystem components, whereas Cluster 40 entails DEGs that mediate translational control and carbon-starvation responses, indicating adjustments in light-harvesting capacity under stress and prolonged energy deprivation. Interestingly, the DEGs of Cluster 33 (degradation of branched-chain amino acids (valine, leucine, and isoleucine)) and of Cluster 36 (sucrose biosynthesis) reinforce the metabolic reprogramming and cellular resource allocation during stress adaptation to maintain the carbohydrate pools for osmoprotection and energy supply. Finally, the DEGS from Cluster 25 (involved in DNA replication initiation), Cluster 11 (enriched for actin cytoskeleton remodeling), and Cluster 19 (associated with autophagy) reveal structural remodeling as a part of genome stability and intracellular recycling processes.

Subsequently, a PPI network was constructed for the 953 DEGs from Group 4 (microarray, root) meta-analysis (Figure 4B; Supplementary File S1d). Using STRING, 929 genes were recognized, and the network was built with experimentally validated interactions (interaction score ≥ 0.4, minimum strength ≥ 0.01). The resulting network comprised 166 nodes and 259 edges. The clustering with the Markov Cluster Algorithm (MCL; inflation parameter = 2) yielded 36 distinct clusters (Supplementary File S3c,d). Several clusters (Figure 4B) were associated with gene expression regulatory processes, which is in accordance with our findings from the meta-analysis of the RNA-Seq data. Clusters 1 to 6 are linked to chromatin modification, transcriptional regulation, RNA processing, and translation-core processes that likely facilitate rapid reprogramming of stress-responsive gene expression under drought conditions. The DEGs from Clusters 13 to 15 (enriched in mitochondrial ATP synthesis, electron transport, and anion exchangers) support the role of the roots in maintaining energy production under stress. Cluster 16 is associated with desiccation response mechanisms, reflecting a direct involvement in drought tolerance. Interestingly, Cluster 34 is associated with the abscisic acid (ABA) signaling pathway, which is a central regulator of plant growth and environmental stress responses.

To ensure functional consistency across tissues in the microarray meta-analysis, we applied a uniform clustering and annotation approach to the PPI networks (Figure 4). While we previously showed several tissue-specific clusters, our analysis also unveils a conserved core of functional groups that are shared between the seedling/shoot and root. Specifically, translation, chromatin remodeling, mitochondrial energy metabolism, motor proteins, and mRNA splicing stand out as the dominant biological themes in both networks. These shared modules (highlighted by the double-lined red and orange lines in Figure 4) demonstrate that despite physiological differences between tissues, the fundamental machinery for maintaining genomic stability and energy homeostasis remains highly conserved across the entire plant.

### 2.4. Functional Enrichment Analysis of DEGs from RNA-Seq and Microarray Datasets Meta-Analyses

**Figure 5:**
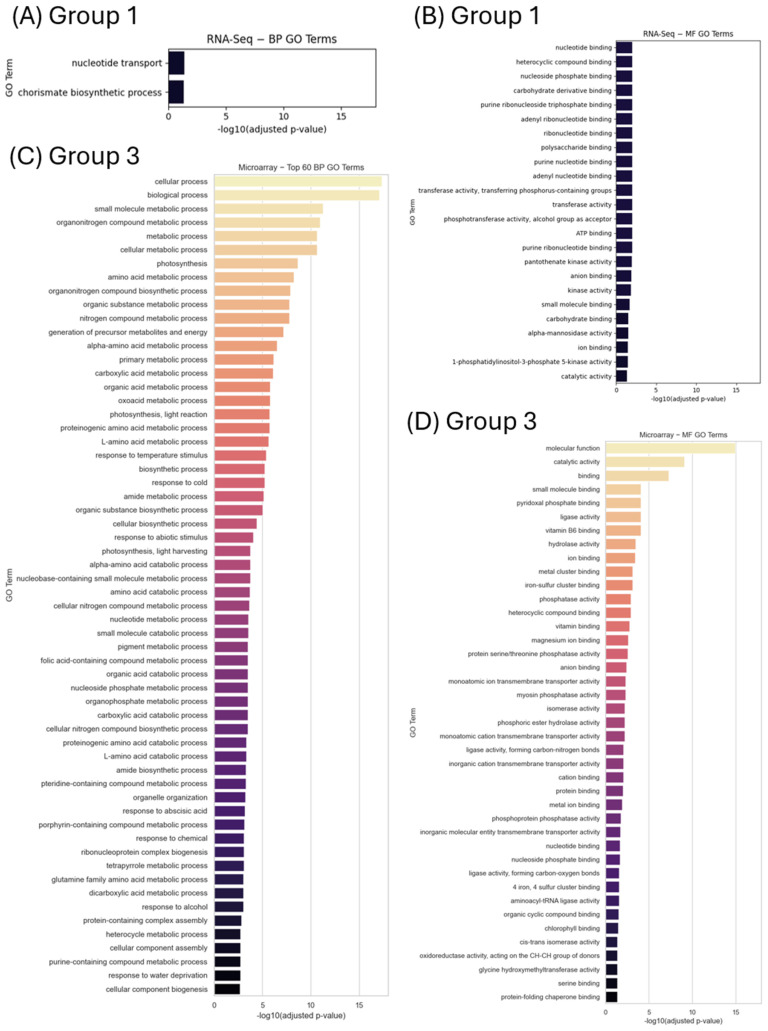

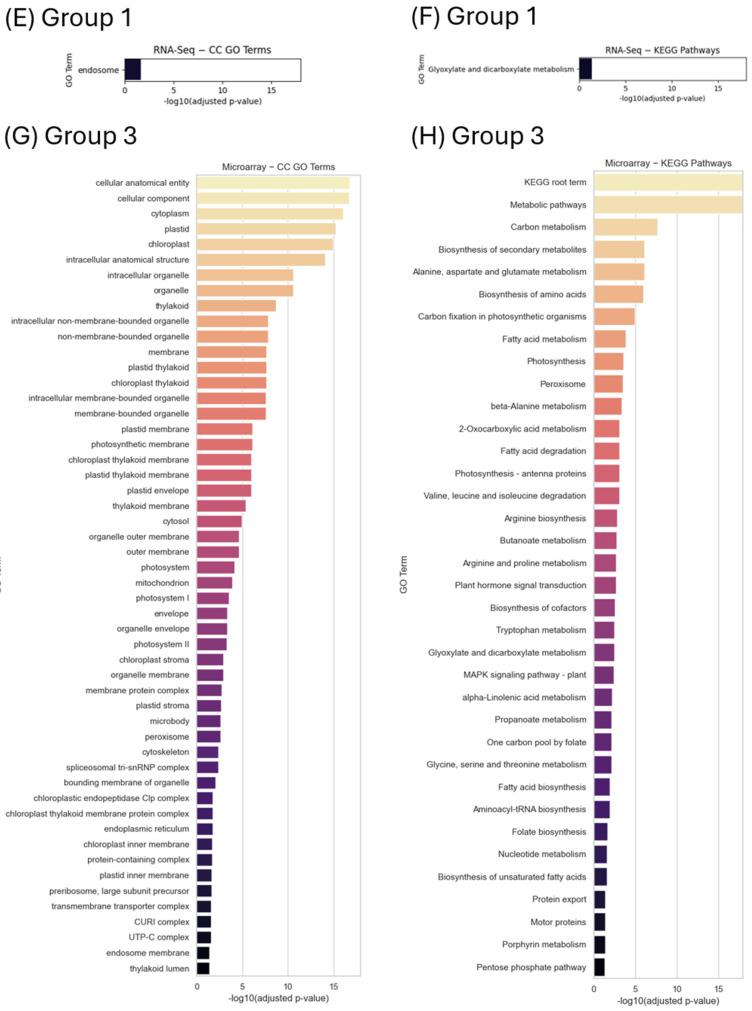
Gene ontology (GO) enrichment analysis by g:Profiler for the DEGs identified from the meta-analyses of: (**A**) the biological processes (BP) from the RNA-Seq seedling/shoot meta-analysis; (**B**) the molecular functions (MF) from the RNA-Seq seedling/shoot meta-analysis; (**C**) the biological processes (BP), top 60, from the microarray seedling/shoot meta-analysis; (**D**) the molecular functions (MF) from the microarray seedling/shoot meta-analysis; (**E**) the cellular compartments (CC) from the RNA-Seq seedling/shoot meta-analysis; (**F**) the KEGG pathways from the RNA-Seq seedling/shoot meta-analysis. (**G**) the cellular compartments (CC) from the microarray seedling/shoot meta-analysis; and (**H**) the KEGG pathways from the microarray seedling/shoot meta-analysis. Color intensity across all charts corresponds to statistical significance (−log10 adjusted *p*-value), with the lighter colors indicating a higher significance.

To further investigate and uncover possible underlying molecular mechanisms, biological pathways, and cellular processes significantly affected or regulated by the DEGs identified through meta-analysis, g:Profiler and PANTHER online tools were utilized. These analyses were additionally used to verify and strengthen the findings obtained from the STRING network analysis and to ensure consistent functional interpretation across complementary bioinformatic platforms. From the 578 DEGs seedling/shoot RNA-Seq data meta-analysis (Group 1), g:Profiler recognized 479 DEGs while PANTHER recognized 450 DEGs. Only g:Profiler provided 27 enriched GO terms (comprehensively BP, MF, CC, and KEGG), since it is unique in exhaustive pathways/GO mining across many sources [39] (Figure 5A,B,E,F), while no enriched terms were retrieved from PANTHER. Of these 27 GO enriched terms, two were related to biological processes (BP) and included energy- and metabolism-related pathways (Figure 5A, Supplementary File S4A). Twenty-three molecular function (MF) terms encompassed protein stability and function pathways (Figure 5B, Supplementary File S4B). The cellular component (CC)-enriched term was that of the endosome (Figure 5E, Supplementary File S4E), while glyoxylate and dicarboxylate metabolism, recapitulating biosynthesis of carbohydrates from fatty acids, was significantly enriched in KEGG pathways analysis (Figure 5F, Supplementary File S4F).

The recruitment of g:Profiler and PANTHER enrichment analysis tools to examine potentially enriched pathways in the root RNA-Seq DEGs (Group 2) resulted in the recognition of 308 and 277 out of 309 DEGs, respectively. However, no significantly enriched terms were observed.

To further investigate and verify our network-based findings obtained from STRING dB, the functions of the 3167 DEGs that were identified in the microarray seedling/shoot meta-analysis (Group 3), we utilized g:Profiler, PANTHER, and STRING. Consistently, the g:Profiler enrichment analysis of the biological processes (BP) revealed a strong overrepresentation of terms linked to photosynthesis (light harvesting, light reaction, and chlorophyll metabolism). Stress-related processes were also prominent (abscisic acid, water deprivation, osmotic stress, and temperature). Protein biosynthesis and ribosome assembly (translation and ribosome biogenesis) were also significantly enriched (Figure 5C, Supplementary File S4C). The g:Profiler molecular function (MF) analysis included terms associated with ion binding and transmembrane transporter activity, supporting roles in osmotic regulation (Figure 5D, Supplementary File S4D). The cellular component analysis revealed a strong enrichment of chloroplast-associated structures, particularly the thylakoid system (Figure 5G, Supplementary File S4G). The KEGG enriched pathways included photosynthesis and carbon fixation, alongside carbon metabolism, and amino acid biosynthesis (Figure 5H, Supplementary File S4H). The PANTHER enrichment analysis of BP (Supplementary File S5a) showed that the 3167 DEGs are primarily involved in biological processes related to photosynthesis and chloroplast function, stress and hormone responses (including abscisic acid), and protein biosynthesis. The enriched MF DEGs were found to play roles in ion transport, osmoregulation, and energy metabolism, while the CC analysis indicated that most DEGs are expressed in chloroplasts, membranes, and thylakoids.

Finally, functional enrichment analysis was performed for the key pathways of the 953 DEGs from the root microarray meta-analysis (Group 4) using the g:Profiler and PANTHER databases. The g:Profiler-enriched DEGs (Supplementary File S6c) included biosynthetic processes of macromolecules in biological processes. The PANTHER enriched DEGs (Supplementary File S6a) included biological processes related to metabolism, stress-associated responses, and biogenesis. The molecular function enrichment revealed hydrolase activities and metal ion binding terms. The cellular component analysis of the rice root DEGs showed a strong association with intracellular organelles and their membranes.

### 2.5. Comparative Analysis of DEGs Derived from Meta-Analyses of Microarray and RNA-Seq Data Across Seedling/Shoot and Root Tissues

To assess the overlap and divergence in the results obtained through the microarray and RNA-Seq meta-analyses, Venn diagrams were generated for the DEGs and the g:Profiler biological pathways for seedling/shoot tissue, where statistically significantly enriched pathways were obtained (Groups 1 and 3).

**Figure 6:**
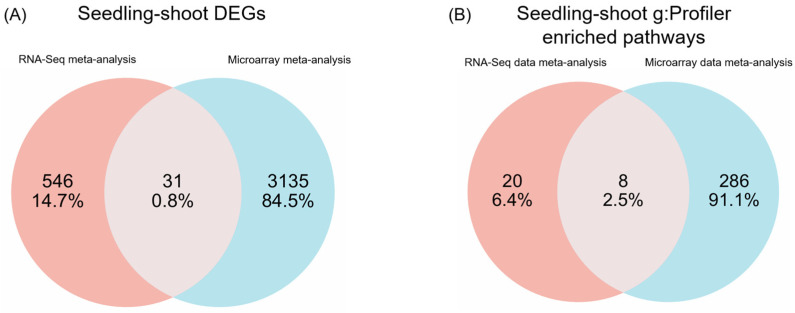
Overlap between RNA-Seq and microarray meta-analysis results in rice seedling/shoot tissue. (**A**) The common differentially expressed genes (DEGs) identified by the RNA-Seq and microarray meta-analyses, and (**B**) the shared enriched biological pathways from the g:Profiler analysis of the 31 common DEGs.

**Figure 7:**
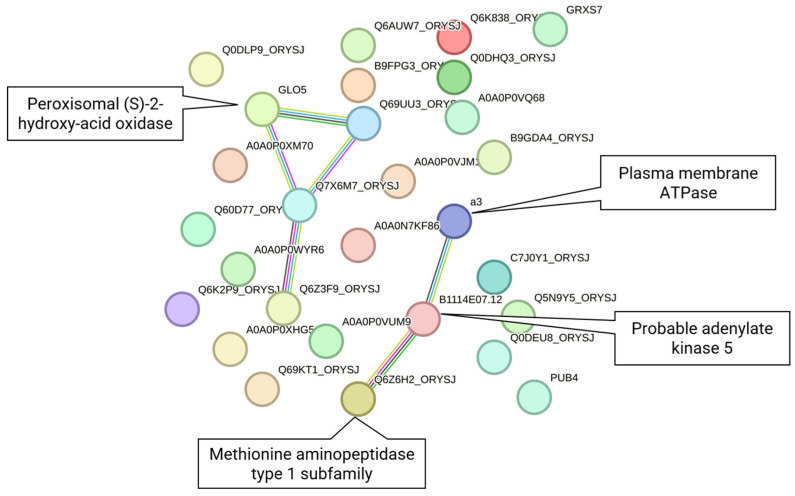
PPI network of the 31 differentially expressed genes (DEGs) common to both the RNA-Seq and microarray seedling/shoot meta-analyses. The edges indicate evidence from STRING, including text mining (yellow), experimental data (magenta), databases (light blue), co-expression (black), neighborhood (green), gene fusion (red), and co-occurrence (blue).

As shown in Figure 6A, while the microarray meta-analysis captured a larger volume of DEGs (3135) compared to RNA-Seq (546), the 31 genes common to both platforms highlight the most conserved and significant transcriptional changes across the integrated datasets. These 31 proteins are involved in chloroplast and organelle membrane function, carbon-related energy turnover, signal transduction, heterocyclic compound binding, ion binding, and transport (Table 6 and Supplementary File S7), reinforcing the importance of these pathways in drought stress adaptation. The PPI network of these 31 DEGs that was constructed in STRING (Figure 7) showed that hub genes with known functions are *GLO5* [Peroxisomal (S)-2-hydroxy-acid oxidase] and B1114E07.12 (probable adenylate kinase 5). Other proteins with annotated functions include Q6Z6H2_ORYSJ (methionine aminopeptidase) and A3 (plasma membrane ATPase).

To determine whether the divergent seedling/shoot DEG sets reflect convergent mechanisms, we intersected the g:Profiler-identified pathways from the RNA-Seq and microarray meta-analyses and summarized the common pathways in a Venn diagram (Figure 6B). This analysis uncovered eight pathways in common, including glyoxylate and dicarboxylate metabolism, nucleotide and heterocyclic compound metabolism, and ion binding (Table 7), which are in high concordance with genes retrieved from the annotation of the 31 common genes. Although only 0.8% of the DEGs were common, 2.5% of pathways overlapped, showing a discrepancy in enrichment analysis, which is expected since even a few shared genes can contribute to multiple interconnected pathways. The overlap between the pathways retrieved from functional annotation and those identified independently with g:Profiler provides a cross-method verification, increasing confidence in the robustness of the results. While not an experimental validation, this consistency demonstrates that both approaches converge on the same biological processes, strengthening their biological relevance.

**Figure 8:**
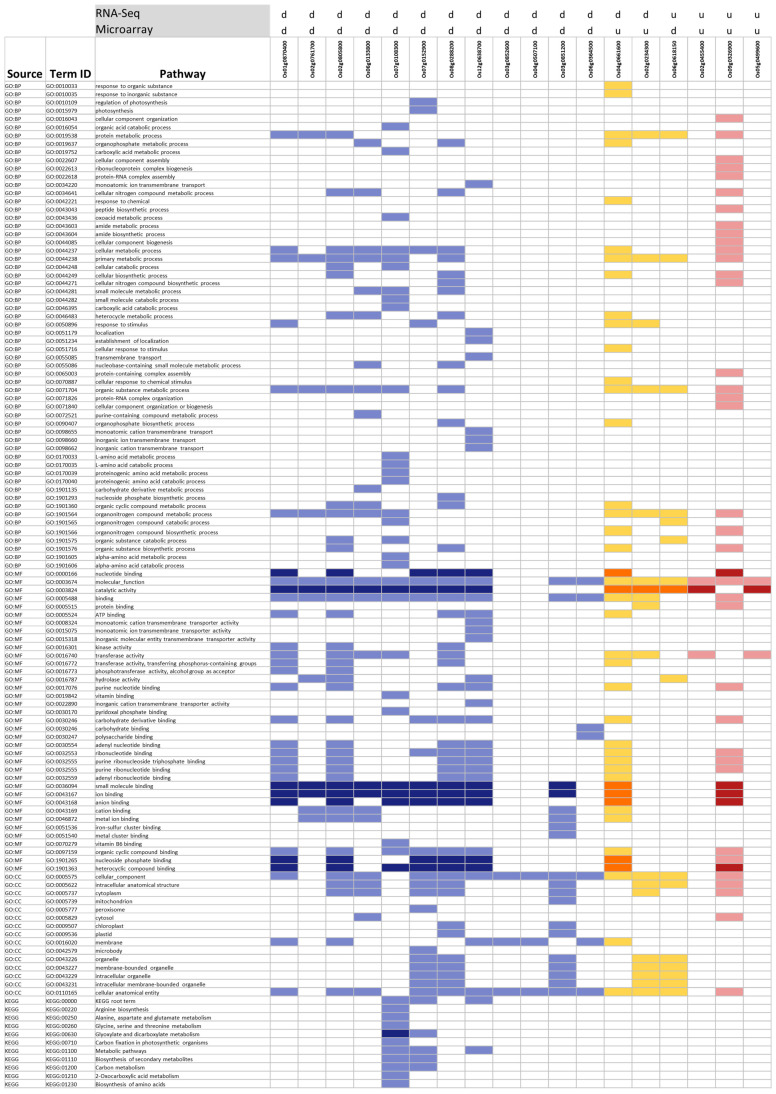
Cross-methodology comparison of the DEGs and their associated enriched pathways in rice seedling/shoot tissue. The x-axis represents shared DEGs, while the y-axis lists enriched pathways identified via g:Profiler. Each cell is color-coded based on the expression pattern and platform-specific pathway enrichment: dark blue–downregulated across both platforms and enriched in the same methodology; blue–downregulated, enriched in the designated pathway in only one methodology; dark orange–differentially expressed (up/down) across both methodologies and enriched in the same pathway; orange–differentially expressed across both methodologies, but enriched in the designated pathway in only one methodology; red–upregulated across both methodologies and enriched in the same pathway; and pink–upregulated, enriched in the designated pathway in only one methodology.

To evaluate whether the 31 DEGs shared between the RNA-seq and microarray seedling/shoot meta-analyses converge on common biology, we mapped them to g:Profiler-enriched pathways (GO:BP, GO:MF, GO:CC, and KEGG) and visualized the overlap in Figure 8. Eighteen of the 31 DEGs were assigned to at least one enriched pathway from either methodology. Of these, 12 showed concordant up-regulation across both analyses (blue), three showed concordant down-regulation (red), and three displayed discordant directionalities between platforms (orange). Eight pathways were enriched in both meta-analyses and are highlighted in dark shades (dark blue, dark orange, and dark red). Figure 8 aligns the 18 DEGs with their corresponding enriched pathways, emphasizing the candidates most indicative of drought stress based on their recurrence across methods.

For the root tissue DEGs, five DEGs were found to be shared between the microarray and RNA-Seq meta-analyses (Groups 2 and 4): *Os02g0782300*, *Os03g0196000*, *Os03g0745600*, *Os09g0106700*, and *Os09g0482720* (*Q8H7X4_ORYSJ*). However, no common significantly enriched pathways were recorded since the RNA-Seq-derived DEGs failed to reach statistical significance in any pathway enrichment analysis. Therefore, no further illustrations are shown for pathway comparisons.

## 3. Discussion

As climate change increasingly threatens rice yields, there is a growing urgency to develop drought-tolerant varieties. This necessity has driven a massive production of multi-omics data, offering deeper insights into how the rice plant manages physiological and metabolic stress [40,41,42,43]. In this landscape, meta-analysis emerges as a powerful tool to synthesize heterogeneous data and provide robust associations leading to critical, condition-dependent tissue-specific insights [19,44]. In the present study, large-scale transcriptomics data were explored to identify genes that are differentially expressed between drought and normal rice samples. The meta-analyses of RNA-Seq data identified 578 and 309 DEGs that were significantly linked to drought in seedling/shoot and root tissues, respectively. Similarly, the meta-analyses of microarray data revealed 3167 and 953 DEGs that were significantly associated with drought in seedling/shoot and root tissues, respectively. The DEGs were identified using a consistent t-test framework and a stringent false discovery rate threshold (FDR < 0.001), allowing for a comparison between the two technologies.

The PPI network construction for the DEGs that were identified from the RNA-Seq seedling/shoot (Group 1) meta-analysis identified a limited but biologically meaningful set of interactions involving genes associated with critical biological pathways such as the DNA helicase complex, structural constituents of ribosomes linked to translational control, and plant mitochondrial respiratory complex. These findings are in line with results from the g:Profiler enrichment analysis, which highlighted pathways related to energy metabolism (BP), protein stability (MF), and glyoxylate and dicarboxylate metabolism (KEGG). Importantly, our computational findings reinforce and independently verify observations from experimental studies showing that these processes are fundamental to early plant development and to the physiological and metabolic response of rice to drought stress [45,46,47,48,49,50]. Biochemical and expression studies have shown that glyoxylate reductases function in the detoxification of aldehydes during stress and contribute to redox balance [51,52], findings that are in accordance with our results, where glyoxylate and dicarboxylate metabolic pathways (g:Profiler) are enriched, suggesting that enhanced photorespiratory activity and energy conservation mechanisms do operate under stress conditions. A closer examination of the DEGs that were identified herein uncovers an interconnectivity of the above pathways. For example, Os09g0326900 is a protein involved in the ribosomal system but is also linked to responses to phytohormones and abiotic stresses [53]. Another protein, Os06g0604500, involved in the plant mitochondrial respiratory complex processes, has previously been proposed as a glucose signal transduction mediator for controlling osmotic stress in rice [54]. The rice phosphoribulokinase gene, *Os02g0698000*, has also been reported to be associated with low temperature abiotic stresses [55,56].

The STRING analysis showed that the 3167 DEGs from the seedling/shoot microarray meta-analysis (Group 3) are involved in photosynthesis, translational control, sucrose biosynthesis, hormone signaling, and stress-response pathways [57,58,59]. These findings are in line with starch degradation and carbon-starvation responses, which are procedures that have been shown to be key regulators of carbon and energy balance under drought conditions [60,61,62,63,64]. The STRING enrichment analysis highlighted photosynthesis-related BPs, ion transport and binding (MF), chloroplast-associated components (CC), and KEGG pathways that were linked to amino acid and carbohydrate metabolism (Supplementary Figure S1A–D). These observations are strongly supported by pathway enrichment analyses (g:Profiler and PANTHER), which reveal a significant overrepresentation of genes involved in photosynthesis, ABA and osmotic stress responses, and protein biosynthesis.

While the Group 2 (RNA-Seq) root DEGs showed limited interactions and no enriched pathways, they included important genes like *Os07g0497100* (crown root formation [65,66]) and others involved in transcriptional regulation and ER protein degradation. The lack of enrichment, which is concordant with the Group 4 microarray data, reflects the limited and dispersed nature of root metabolic responses compared to other plant tissues. Biologically, this suggests that the root drought response is characterized by broad, subtle regulatory adjustments across many functional categories rather than a massive metabolic shift in a single pathway. This is in accordance with findings of others [67], where the root DEGs are spread across diverse families rather than clustering in primary metabolic pathways [68]. Furthermore, the roots’ enriched pathways are dominated by transcriptional DEGs (Supplementary Figure S2), which is in accordance with findings from Özmen et al., who showed that transcription factors trigger shallow changes across many pathways, diluting statistical signals [69]. Moreover, genotype-specific variability and incomplete annotation of root-specific pathways could have masked common stress signatures [69]. Despite these limitations, the STRING, PANTHER, and g:Profiler analyses in root tissue, besides transcription, uncovered broader overrepresentation of the DEGs in metabolism, development, and stress response, specifically within intracellular membranes and catalytic binding activities (Supplementary File S6a,c).

While our findings align with previous QTL- and haplotype-based meta-analyses regarding core molecular pathways that include ribosomal function, RNA-mediated translational control, ER-regulated protein degradation, and hormone (e.g., ABA) signaling [70,71,72], this study identifies additional regulatory mechanisms that were previously undetected in meta-analytical contexts. Specifically, we revealed a significant role for glyoxylate and dicarboxylate metabolism in carbohydrate and lipid biosynthesis, probably acting to mitigate carbon-starvation-induced pathways resulting from reduced photosynthetic activity. Furthermore, specialized transport systems for ions, electrons, and heterocyclic compounds were found to be actively engaged across organelle membranes, specifically within the mitochondria, endosomes, and chloroplast thylakoids. Finally, our results highlight the importance of autophagy-related genes and actin-dependent cytoskeleton remodeling, suggesting a complex structural and morphological adaptation to drought stress.

In our meta-analyses, the larger DEGs set that was obtained from the microarrays meta-analysis compared to RNA-seq is likely attributable to differences in the chemistry principles and computational sensitivity between platforms and methodologies. The substantially higher number of DEGs identified in the microarray meta-analysis compared to RNA-Seq likely reflects differences in data structure and preprocessing. The microarray intensity data are continuous and often exhibit lower inter-study variance after standardized normalization, which can artificially inflate statistical power and lead to higher DEG yields in a meta-analysis. Furthermore, microarrays are limited by probe-target hybridization kinetics and a narrower dynamic range compared to RNA-Seq. In contrast, the RNA-seq datasets are affected by between-study variability in sequencing depth, library preparation, and low-count gene distributions, all of which interact with dispersion modeling and normalization algorithms to produce more conservative FDR-adjusted significance estimates [65,66,73,74,75,76,77]. Furthermore, the precision of the DEG detection in the RNA-Seq pipelines can be significantly enhanced by specialized data-cleaning protocols. For instance, the recently developed RNAdeNoise [78] tool utilizes statistical modeling to identify and remove technical noise, thereby improving the detection of differentially expressed genes with low-to-moderate transcription levels without the subjectivity of fixed count thresholds. While our study utilized a standardized TPM-based framework for cross-study normalization, adopting such de-noising methodologies represents a promising avenue for refining stress signatures in future large-scale meta-transcriptomic analyses. Therefore, the difference in the DEG numbers reflects the combined influence of biological variability, data structure, dynamic range, and pipeline-dependent statistical behavior rather than mere intrinsic platform superiority, aligning with observations reported in multiple benchmarking studies.

In our effort to synthesize and compare results across the two different transcriptomics methodologies for Group 1 and Group 3 (seedling/shoot meta-analyses) DEGs (578 and 3167), we further studied their 31 common genes (Figure 6A and Figure 7, Table 6). The pathway analysis using g:Profiler across both methodologies further revealed eight commonly enriched pathways between the two methodologies (Figure 6B and Figure 8, Table 7). Although only 5.4% of the RNA-Seq DEGs overlap with those from microarrays, 29% of the RNA-Seq-enriched pathways are shared with the microarray-enriched pathways, indicating that both methods can identify key representative biological pathways (Figure 6A,B). These 31 shared proteins have been previously shown to play pivotal roles in rice drought stress response (Table 6). The receptor-like kinase Os01g0870400 belongs to the RLK family, which mediates abiotic stress signaling in plants [79]. GLO5 (Os07g0152900), a glycolate oxidase isozyme, participates in photorespiration [80]. Adenylate kinase 5 (Os08g0288200), also chloroplast-localized, may support energy homeostasis, though its specific function in rice remains unclear. OsAHA3 (Os12g0638700), a plasma membrane ATPase, is linked to saline–alkaline stress tolerance [81]. GRXS7 (Os03g0851200), a chloroplast-localized glutaredoxin, contributes to redox homeostasis under drought stress [82]. Interestingly, three genes display opposing expression patterns between the two methodologies, either upregulated or downregulated, yet converge on similar functional pathways (Figure 8). The molybdenum cofactor biosynthesis gene (*OsCNX1*, alias *Os04g0661600*), down-regulated in RNA-Seq and up-regulated in microarray meta-analysis, is crucial for proper seed germination and seedling development in rice [83]. The E3 ubiquitin ligase PUB4 (*Os02g0234300*), an *Arabidopsis thaliana* ortholog, found upregulated in microarray in contrast to RNA-Seq meta-analysis, is thought to participate in circadian rhythms and abiotic stress responses [84] and may influence immunity and seedling development [85]. Several other proteins and non-coding transcripts (e.g., Os02g0455400 and Os03g0210900) lack functional characterization under drought conditions and require further investigation.

The shared enriched pathways derived from the comparison of RNA-Seq and microarray meta-analyses (Groups 1 and 3) highlight key biochemical processes involved in drought adaptation of rice seedling/shoots (Table 7). Although limited in number, these pathways consistently point to ABA-mediated hormonal regulation, ROS detoxification, osmotic adjustment, the maintenance of photosynthetic and energy balance, and ion homeostasis. Notably, glyoxylate and dicarboxylate metabolism emerges as a novel mechanism supporting energy conservation and reactive oxygen species scavenging under stress. Thus, the combined meta-analysis across datasets provided a deeper insight into tissue-specific drought responses. The seedling/shoot DEGs (Groups 1 and 3) predominantly reflected photosynthetic and carbon fixation and metabolic adjustments, translational control, phytohormone signaling, and membrane-conjugated small molecule transport, whereas the root DEGs (Groups 2 and 4) were enriched for chromatin remodeling, transcriptional and translational regulation, metabolic and growth-related pathways, ABA signaling, and diverse molecular binding and transport activities related to osmotic stress adaptation. Together, these patterns demonstrate functional divergence between tissues (photosynthetic adjustment in shoots versus regulatory and metabolic reprogramming in roots). Yet, these patterns converge on common core drought-response mechanisms such as ABA signaling, ion binding and membrane transport, and translational control.

Comparison of our findings with previous transcriptomic meta-analyses in rice reveals both strong biological agreement and important methodological differences. The RNA-Seq meta-analysis by Derakhshani et al. [86] examined drought responses across ten tolerant and three susceptible varieties using mixed tissues (leaf, root, shoot, stem, inflorescence, and panicle) and multiple developmental stages, aiming to define a unified drought-responsive gene set rather than resolve tissue-specific signatures. Similarly, the microarray-based drought meta-analysis by Sirohi et al. [87] incorporated only three datasets (two root studies and one whole-seedling dataset), offering limited resolution for cross-tissue comparisons. Singh et al. [88] focused on heat stress, analyzing seedlings, leaves/flag leaves, and panicles at reproductive stages, and therefore did not investigate drought responses or root–shoot contrasts. Despite the differences in scope, all three studies identified photosynthesis, carbohydrate metabolism, ABA signaling, ROS detoxification, chromatin remodeling, and transcriptional regulation as key pathways, all of which align with the stress mechanisms identified in our analyses. While the Bayesian integration framework of Ma et al. [89] offers a probabilistic approach for combining microarray and RNA-Seq data, our study adopts a direct two-stage workflow that preserves methodological transparency and enables a clear tissue-specific comparison across platforms. This dual-platform two-tissue framework provides a more symmetric and biologically interpretable assessment than previous analyses of mixed or single tissues and represents the first integrated evaluation of drought-responsive transcriptomic pathways in wild-type rice spanning across both microarray and RNA-Seq technologies. Moreover, our ability to detect shared pathways, even when the overlapping of DEGs is limited, underscores the strength of cross-platform synthesis in revealing robust biological signals that might be obscured in platform-specific analyses. Together, these observations highlight that integrating tissue-resolved microarray and RNA-Seq meta-analyses not only validates core drought-responsive mechanisms that have been identified across independent studies but also offers a more comprehensive and balanced understanding of stress adaptation in rice. By integrating methodological and tissue-specific datasets, our work establishes a unified framework for cross-platform transcriptomic synthesis and provides a solid foundation for future functional studies targeting drought-resilient traits.

Our stratified meta-analysis of rice drought responses is subject to several limitations arising from the diversity and complexity of publicly available datasets. First, the limited number of drought-stressed and corresponding control wild-type rice samples necessitated the grouping of seedling and shoot datasets into a single tissue category to achieve sufficient statistical power, while roots were analyzed separately. Although this approach improves robustness, it may mask finer developmental-stage effects. Second, substantial heterogeneity across studies (including differences in growth conditions, drought severity and duration, sampling time points, plant age, cultivar background, and tissue collection protocols) introduces a variability that cannot be fully corrected by random-effects modeling. These discrepancies, together with the platform-related batch effects and unequal sequencing depths, have likely contributed to the differences in the DEG numbers between the RNA-Seq and microarray datasets. Third, the metadata incompleteness (e.g., information on environmental conditions, nutrient status, irrigation regimes, or physiological measurements) made it difficult to interpret the transcriptional changes in a fully comparable context. Additionally, aligning the gene identifiers across the studies posed substantial challenges due to inconsistencies between the RAP-DB and RGAP annotations. The preferential use of RAP-DB by most current functional tools increases the difficulty of handling the datasets that were annotated with RGAP identifiers [90]. Notably, the scarcity of root-specific transcriptomic datasets represents a significant constraint. This data sparsity likely limited the statistical power of our root-tissue analysis, potentially explaining the lack of statistically significant gene associations in the STRING networks and the inability to identify enriched functional pathways for this tissue. Finally, despite careful searching, we cannot exclude a degree of publication bias. Altogether, these limitations highlight the need for better standardized, well-annotated, multi-tissue, developmentally staged, drought-scenario-reported transcriptomics datasets to enable more refined and reliable meta-analysis.

Even with these limitations, our study provides a broad and integrative picture of how rice responds to drought at the transcriptional level. The directional inconsistencies across datasets were addressed using the random-effects model, which incorporates between-study heterogeneity into the weight calculation; genes with highly conflicting expression patterns across studies resulted in non-significant pooled effect sizes. By combining the RNA-Seq and microarray data and explicitly separating seedling/shoot and root tissues, we were able to unravel shared and tissue-specific components of the drought response and identify the gene modules and pathways that persist across the platforms. The recurring signatures we observed (adjustments in photosynthesis and carbon metabolism, ABA signaling, ROS detoxification, chromatin remodeling, and translational control) and the newly uncovered mitochondria-related energy transport and small molecule membrane transport, and the response to carbon starvation via involving biosynthesis of carbohydrates or fatty acids from two-carbon precursors point to core mechanisms that are likely central to drought adaptation in rice. The gene candidates highlighted here (e.g., *Os07g0152900 (GLO5)*: glyoxylate and dicarboxylate metabolism, *Os03g0851200 (GRX)*: Glutaredoxin S7 involved in redox signaling, or *Os02g0234300 (PUB4)*: plant U-box E3 ubiquitin ligase involved in various hormone signaling pathways), including both well-characterized stress regulators and poorly annotated transcripts, provide concrete starting points for future work using functional genomics approaches such as CRISPR-based editing, overexpression, and tissue-specific reporter analyses. As climate change continues to increase the frequency and severity of water limitation, increasing our understanding of these pathways in diverse genetic backgrounds and under field-relevant conditions will be essential to translating the transcriptomics insights into durable improvements in rice resilience and productivity.

## 4. Materials and Methods

### 4.1. Data Extraction

Using the preferred reporting items for systematic reviews and meta-analyses (PRISMA) statement [91], two comprehensive searches of the literature were conducted by three investigators (M.K., K.M. and A.G.) independently in the Gene Expression Omnibus (GEO) database [92] in December, 2023, to extract all the available RNA-Seq and microarray datasets in relation to drought stress in rice. The terms “Oryza sativa” and “Oryza sativa” [porgn:_txid4530], along with additional filters “Expression profiling by high throughput sequencing” and “Non-coding RNA profiling by high throughput sequencing” led to the retrieval of datasets for the RNA-seq (M.K., K.M.) and microarray (M.K., A.G) experiments, respectively. The selection criteria demanded that the selected RNA sequencing or microarray studies only reported gene expression data on healthy root, shoot, and seedling tissues from wild-type (*indica* or *japonica*) rice plants that had either normal treatment (control plants) or had undergone various types of drought or desiccation stress, e.g., dehydration with PEG, limited water supply ranging from 20% to 10% soil water content (case plants). The excluded studies were those that utilized non-NGS-based RNA-Sequencing or non-microarray approaches; tested tissue samples from genetically modified plants or transgenic rice lines; reported data from rice tissue other than the ones reported above, such as leaf, panicle, or another; or included less than 5000 unique records of genes in their final gene expression dataset.

### 4.2. Data Preprocessing

The RNA-seq transcriptomics data of the selected studies were obtained directly from the Supplementary Files in the GEO database. Because in almost all the datasets the gene nomenclature system used was according to the Rice Genome Annotation Project (RGAP) [93,94], this annotation system was retained across all the RNA-Seq data matrices by converting all the other gene names [from Rice Annotation Project Database (RAP-DB) [95]] into RGAP 7.0.

According to the RGAP, each nuclear-encoded gene is labeled LOC_OsXXg#####, with LOC_Os referring to Oryza sativa locus, XX referring to chromosome 01–12, g referring to gene, and a 5-digit number referring to the gene order on the chromosome. The Rice Annotation Project Database (RAP-DB) is an international rice genome sequencing project assembly that uses an ID (OsXXg#######) consisting of the species name (Os for Oryza sativa), a two-digit number for chromosomes, and a seven-digit number that indicates a sequential order of loci in a chromosome. These Os identifiers are different from the ones that are used by the RGAP; thus, the gene/locus names according to RAP-IDs were converted to RGAP locus IDs using the RAP-DB ID converter tool (https://rapdb.dna.naro.go.jp/converter/, accessed on 15 April 2024).

The RNA-Seq data files contained metrics such as TPM (transcripts per million) [96] and RPKM/FPKM (Reads/Fragments Per Kilobase of transcript per Million mapped reads) [97,98]. To ensure that there was consistency and comparability across datasets, all the RNA-Seq gene expression data were converted to transcripts per million (TPM) values. The TPM values have been shown to meet the invariant average criterion and thus are more comparable across samples [99]. The conversion of RPKM/FPKM values to TPM was performed using the following formulas:

(1) $${\;TPM}_{i}=\frac{{FPKM}_{i}}{\sum_{j}{FPKM}_{j}}\times 10^{6}$$

(2) $${TPM}_{i}=\frac{{RPKM}_{i}}{\sum_{j}{RPKM}_{j}}\times 10^{6}$$

The genes containing zero reads across every sample in a single study were filtered out prior to the data analysis. Concerning the datasets from the microarray studies, the series matrix files were downloaded from each relevant GEO dataset entry. The probes were also transformed into RGAP gene IDs, as suggested by Ramasamy and their coworkers [100]. When several probes had mapped to the same gene, their signal intensities were averaged to eliminate the probe-to-gene many-to-many overlap, yielding a single mean expression value for each gene.

To maintain methodological integrity, we analyzed the RNA-Seq and microarray data through separate, independent meta-analyses rather than combining them into a single pool. This ‘dual-track’ approach allowed us to identify robust, platform-independent biological signals by comparing the DEGs and pathways only after the primary statistical analyses were complete.

### 4.3. Statistical Analysis

The datasets from rice plants in control and drought condition were divided into four main groups: (i) Group 1: the RNA-Seq studies on rice seedling/shoot tissue, (ii) Group 2: the RNA-Seq studies on rice root tissue, (iii) Group 3: the microarray studies on rice seedling/shoot tissue, (iv) Group 4: the microarray studies on rice root tissue. Statistical analysis across every individual study was also performed to identify differentially expressed genes (DEGs) between drought and normal tissues. A random effects model meta-analysis was conducted across all four dataset groups [37,101]. While the fold change (FC) is a standard metric for individual transcriptomic experiments, it has significant limitations in a meta-analysis framework because it fails to account for sample size and variance across the different studies. Consequently, the standardized mean difference (SMD) was employed as the effect size metric [102]. We utilized Hedges’ g as the unbiased estimator of the SMD, as it corrects for the upward bias inherent in Cohen’s d for small sample sizes [103,104]. In this context, an effect size of one indicates that the mean difference between the treatment and control equals one pooled standard deviation [19,105]. Each study was weighted by its precision and determined by the inverse of the variance to ensure a robust estimation of the overall effect across all the datasets. Finally, the statistical heterogeneity was evaluated using Cochran’s *Q* test [106], while the magnitude of heterogeneity and the between-study variance were quantified using the *I*^2^ statistic [107] and *τ*^2^ [108], respectively.

To address the issue of multiple comparisons, this study considered various correction methods that were categorized into two groups: those that control family-wise error rate (FWER) and those that control false discovery rate (FDR). The Bonferroni correction [109] is the most commonly used approach for controlling FWER. It is straightforward and intuitive but often regarded as overly conservative. Other widely used methods for FWER correction include those developed by Sidak [110], Holland and Copenhaver [111], and Holm [112]. In contrast, the method proposed by Benjamini and Hochberg [113] controls FDR rather than FWER. The FDR-controlling methods aim to regulate the expected proportion of incorrectly rejected null hypotheses among all rejections [113]. These methods offer greater statistical power at the expense of a higher likelihood of Type I errors by enabling the identification of more significant differences. To determine the optimal stringency for our analysis, we conducted a comparative sensitivity analysis across a range of FDR thresholds (from 0.05 to 0.0001) for both RNA-Seq and microarray platforms. This empirical evaluation allowed us to assess the stability of our gene lists and the biological relevance of the resulting enrichment. We observed significant differences in the DEG yields between platforms; for instance, at an FDR < 0.001, the microarray meta-analysis identified a higher volume of significant genes (3167) compared to RNA-Seq (578). Notably, both datasets exhibited a similar ‘cliff effect’ at thresholds that were stricter than 0.001, where individual studies (e.g., GSE128495 in RNA-Seq and GSE79212 in microarray) failed to return any significant DEGs. Selecting FDR < 0.001 as our standard threshold allowed us to maximize the inclusion of independent studies while maintaining a high statistical stringency. All the statistical analyses were performed using MAGE (ver. 2.0) [114], which is a Python-based web tool that was recently developed by our group.

### 4.4. Functional Enrichment Analysis

To identify and characterize the biological processes and biochemical pathways in which the differentially expressed genes (DEGs) are actively involved, we mapped these genes to specific cellular functions, molecular mechanisms, and interaction networks by employing the STRING (Search Tool for the Retrieval of Interacting Genes) [115], PANTHER (Protein Analysis Through Evolutionary Relationships) [116,117] and g:Profiler [39] webtools. The STRING database v12.0 was employed to construct protein–protein interaction (PPI) networks and retrieve all available gene ontology (GO)-enriched terms, as well as KEGG (Kyoto Encyclopedia of Genes and Genomes)-enriched pathways of the DEGs. To ensure high biological stringency, PPI networks were constructed using exclusively experimentally determined interactions. A medium confidence score of 0.4 was applied to balance this rigor with the inherent scarcity of validated rice PPI data, thereby preventing the loss of biologically significant interactions. To reduce large network complexity and extract functional modules, the Markov Cluster Algorithm (MCL) [118], an unbiased clustering algorithm for graphs implemented in STRING, was applied, with an inflation parameter of 2, to identify clusters of similar proteins. This algorithm uses a Markov chain that simulates the flow within a network to identify groups of nodes with high interconnectivity, which indicates potential functional relationships [118,119].

To further analyze protein domains and interaction patterns, particularly in cases where PPI network information was sparse, we utilized domain data from SMART (Simple Modular Architecture Research Tool), which is a database integrated into STRING for identifying and annotating protein domains and analyzing protein domain architectures [120]. Also, the functional annotations from STRING were recorded to generate a functional profile for each protein when the PPI network data were limited or insufficient for drawing conclusions.

The PANTHER database v18.0 was employed to identify enriched GO terms for biological processes (BP), molecular functions (MF), and pathways that the DEGs are involved in. The GO terms retrieved from statistically significant overrepresentation tests at the FDR threshold < 0.05 were considered significant. The g:Profiler web tool was also recruited for enrichment analysis of the DEGs that are associated with enriched GO terms and KEGG pathways. The Benjamini-Hochberg threshold was chosen as the significance threshold, and the user threshold was set to *p* < 0.05.

For the DEGs to be widely recognized by all three web tools, the gene symbols were converted into RAP locus IDs using the RAP-DB ID converter tool prior to the functional enrichment analysis step. For the functional annotation of selected proteins, the UniProt [121], RAP-DB [95], and SMART [120] databases were recruited.

## 5. Conclusions

By statistically integrating independent transcriptomic data, this meta-analysis identifies robust signatures of drought resilience in rice. Rather than nominating a single ‘master regulator’, our findings reveal a sophisticated network of tissue-specific adaptations. Beyond validating core signals like ABA and redox balance, this study uncovers a previously undetected metabolic pivot in the glyoxylate and dicarboxylate metabolism to mitigate carbon starvation. These prioritized gene sets, specifically those involved in organelle-specific transport and actin-remodeling, offer clear targets for functional validation via CRISPR/Cas9 or as biomarkers in marker-assisted breeding programs. While our findings are limited by the inherent heterogeneity of public datasets and the relative scarcity of root-specific studies, this integrative synthesis provides a robust roadmap for targeted biotechnological efforts. Coupling this cross-platform evidence with functional validation can provide a key framework to accelerate the development of drought-resilient rice cultivars, which is essential for securing global food productivity in an era of climatic volatility.

## Figures and Tables

**Table 1:** The RNA-Seq study characteristics included in the present meta-analysis.

Author	GEO Dataset	Publication Year in GEO	Platform	Rice Tissue	*Oryza sativa* Subspecies and Cultivar	Number of Samples Control/Drought	Control Samples	Drought Samples	Conditions Applied on Drought Sample	Number of Unique Genes per Study ^†^	Number of Genes After Processing ^††^
Chen et al. [24]	GSE160679	2021	GPL28757 Illumina HiSeq 3000, Illumina, Inc., San Diego, CA, USA	Seedling	Japonica, Nipponbare	3/2	GSM4876801, GSM4876802, GSM4876803	GSM4876804, GSM4876805	Dehydration treatment with 20% PEG 8000 solution.	34,121	33,021
Sevanthi et al. [25]	GSE147158	2020	GPL25149 Illumina HiSeq 1500, Illumina, Inc., San Diego, CA, USA	Shoot	Indica, Nagina 22 and IR64	2/2	GSM4419428, GSM4419436	GSM4419432, GSM4419440	Drought stress on the 15th day by limiting water supply, 10 mL versus 100 mL water supply, until the 21st day.	37,998	30,790
Ma et al. [26]	GSE117047	2019	GPL13160 Illumina HiSeq 2000, Illumina, Inc., San Diego, CA, USA	Seedling	Japonica, Zhonghua 11 (ZH11)	3/6	GSM3267749, GSM3267750, GSM3267751	GSM3267752, GSM3267753, GSM3267754, GSM3267755, GSM3267756, GSM3267757	Moderate drought stress (20% soil water content) and severe drought stress (10% soil water content).	55,986	38,963
Li et al. [27]	GSE128495	2019	GPL23876 HiSeq X Ten, Illumina, Inc., San Diego, CA, USA	Seedling	Japonica, Zhonghua 11 (ZH11)	3/3	GSM3677682, GSM3677683, GSM3677684	GSM3677679, GSM3677680, GSM3677681	Drought stress treatment by withholding water for a period of 10–15 days.	55,490	30,998
Fu et al. [28]	GSE107425	2018	GPL13834 Illumina HiSeq 2000, Illumina, Inc., San Diego, CA, USA	Shoot	Japonica, Zhonghua 11 (ZH11)	3/2	GSM2866845, GSM2866846, GSM2866847	GSM2866848, GSM2866849	Drought stress treatment at the 4-leaf stage until the water content of the soil is maintained at 10–15%.	55,689	33,889
Yang et al. [29]	GSE136373	2020	GPL23876 HiSeq X Ten, Illumina, Inc., San Diego, CA, USA	Root	Japonica, Zhonghua 11 (ZH11)	3/3	GSM4047572, GSM4047573, GSM4047574	GSM4240719, GSM4240720, GSM4240721	Drought stress for 3 h before sampling.	55,986	32,068
Sevanthi et al. [25]	GSE147158	2020	GPL25149 Illumina HiSeq 1500, Illumina, Inc., San Diego, CA, USA	Root	Indica, Nagina 22 and IR64	2/2	GSM4419427, GSM4419435	GSM4419431, GSM4419439	Drought stress on the 15th day by limiting water supply, 10 mL versus 100 mL water supply, until the 21st day.	37,998	32,030

^†^ The total number of unique genes within the data set. ^††^ The total number of unique genes after processing the data set by filtering out zero elements across all samples.

**Table 2:** The microarray study characteristics included in the present meta-analysis.

Author	GEO Dataset	Publication Year in GEO	Platform	Rice Tissue	*Oryza sativa* Subspecies and Cultivar	Number of Samples Control/Drought	Control Samples	Drought Samples	Conditions Applied on Drought Sample	Number of Unique Probes per Study ^†^	Number of Genes After Processing ^††^
Ogawa et al. [30]	GSE115826	2021	GPL6864 Agilent-015241 Rice Gene Expression, Agilent Technologies, Palo Alto, CA, USA	Shoot	Japonica, Nipponbare	4/4	GSM3190931, GSM3190932, GSM3190933, GSM3190934	GSM3190935, GSM3190936, GSM3190937, GSM3190938	Two-week-old seedlings were subjected to drought conditions.	45,151	29,523
Bhattacharjee et al. [31]	GSE79212	2017	GPL21593 Affymetrix Rice (US) Gene 1.0 ST Array, Affymetrix, Inc., Santa Clara, CA, USA	Seedling	Indica, Pusa Basmati 1	3/3	GSM2088150, GSM2088151, GSM2088152	GSM2088156, GSM2088157, GSM2088158	3-week-old seedlings were dried between folds of tissue paper at 28 ± 1 degree Celsius for 3 h.	54,922	38,588
Borah et al. [32]	GSE41647	2015	GPL2025 Affymetrix Rice Genome Array, Affymetrix, Inc., Santa Clara, CA, USA	Seedling	Indica, Dagad deshi Indica, IR20	6/12	GSM1020924, GSM1020925, GSM1020926, GSM1020927, GSM1020928, GSM1020929	GSM1020930, GSM1020931, GSM1020932, GSM1020933, GSM1020934, GSM1020935, GSM1020936, GSM1020937, GSM1020938, GSM1020939, GSM1020940, GSM1020941	7-day-old seedlings were put on 3 mm Whatman sheets for three hours and six hours at 28 ± 1 °C.	57,381	37,422
Singh et al. [33]	GSE6901	2007	GPL2025 Affymetrix Rice Genome Array, Affymetrix, Inc., Santa Clara, CA, USA	Seedling	Indica, IR64	3/3	GSM159259, GSM159260, GSM159261	GSM159262, GSM159263, GSM159264	7-day-old seedlings were dried between folds of tissue paper at 28 ± 1 degree Celsius for 3 h.	57,381	37,422
Muthurajan et al. [34]	GSE92883	2018	GPL2025 Affymetrix Rice Genome Array, Affymetrix, Inc., Santa Clara, CA, USA	Root	Indica, IR20 Indica, Nootripathu	4/4	GSM2440716, GSM2440717, GSM2440720, GSM2440721	GSM2440718, GSM2440719, GSM2440722, GSM2440723	40 days after sowing, drought stress was imposed by withholding water in the field.	57,381	37,422
Dixit et al. [35]	GSE78504	2016	GPL21470 Agilent-045741 Rice custom expression array, Agilent Technologies, Los Banos, Philippines	Root	Indica, Vandana Indica, IR64	6/6	GSM2071708, GSM2071709, GSM2071710, GSM2071725, GSM2071726, GSM2071727	GSM2071705, GSM2071706, GSM2071707, GSM2071722, GSM2071723, GSM2071724	Drought stress treatment was a drydown from 75% of the field capacity. Tissue samples were collected 25 days after sowing.	52,957	52,900

^†^ The total number of probes in the data set without processing. ^††^ The total number of genes after matching the probes to the genes and removing duplicates.

**Table 3:** The number of statistically significant DEGs identified from the meta-analysis of the RNA-seq datasets for multiple FDRs.

		*p*-Value < 0.05	FDR < 0.05	FDR < 0.01	FDR < 0.005	FDR < 0.001	FDR < 0.0005	FDR < 0.0001
**Seedling/shoot**	GSE117047	17,607	15,468	11,665	10,237	6909	5267	0
	GSE128495	16,290	13,839	9208	6960	0	0	0
	GSE160679	22,314	21,497	18,481	17,185	13,875	12,109	0
	GSE147158	9647	6645	4291	3610	2251	1603	0
	GSE107425	16,225	14,068	10,114	8484	4251	0	0
	**Meta-analysis**	12,956	5078	1663	1248	**578**	398	135
**Root**	GSE136373	20,684	19,131	15,486	13,998	9935	7566	0
	GSE147158	11,032	7507	4522	3590	1986	1492	456
	**Meta-analysis**	6243	2959	1735	1315	**309**	261	27

**Table 4:** The number of statistically significant DEGs identified from the meta-analysis of the microarray datasets for multiple FDRs.

		*p*-Value < 0.05	FDR < 0.05	FDR < 0.01	FDR < 0.005	FDR < 0.001	FDR < 0.0005	FDR < 0.0001
**Seedling/shoot**	GSE41647	17,616	15,293	12,500	11,617	9660	8870	7092
	GSE79212	15,687	10,808	4598	2486	0	0	0
	GSE6901	16,281	13,206	8881	7105	2619	0	0
	GSE115826	23,179	22,911	20,860	20,014	17,947	16,871	13,143
	**Meta-analysis**	15,121	10,418	6365	5124	**3167**	2411	900
**Root**	GSE78504	13,875	6292	2132	1225	0	0	0
	GSE92883	16,583	13,233	8778	7291	3856	1811	0
	**Meta-analysis**	10,260	4184	2194	1713	**953**	731	373

**Table 5:** The integration-driven discovery rate (IDR) and the integration-driven revision rate (IRR) of the meta-analyses. The IDR represents the percentage of DEGs identified through meta-analysis that were not statistically significant in any individual study, highlighting the gain in discovery power. The IRR indicates the percentage of DEGs reported in individual studies that did not reach the significance threshold (FDR < 0.001) in the meta-analysis, reflecting the filtration of study-specific noise and potential false positives.

Methodology	Tissue	Number of Studies	Number of DEGs	IDR (%)	IRR (%)
RNA-Seq	Seedling/shoot	5	**578**	10.6	97.7
	Root	2	**309**	14.0	97.6
Microarrays	Seedling/shoot	4	**3167**	4.5	86.4
	Root	2	**953**	78.7	95.7

**Table 6:** The list of the 31 common (between the RNA-Seq and microarray meta-analysis) seedling/shoot DEGs, with gene identifiers from RAP-DB and corresponding protein product names used in STRING.

Gene ID (RAP-DB)	STRING Protein Node	Protein Description
*Os01g0870400*	Q5N9Y5_ORYSJ	Receptor-like serine/threonine-protein kinase; it belongs to the protein kinase superfamily. Ser/Thr protein kinase family.
*Os02g0234300*	PUB4	U-box domain-containing protein 4; possesses E3 ubiquitin-protein ligase in vitro.
*Os02g0455400*	A0A0N7KF86	Os02g0455400 protein.
*Os02g0518800*	A0A0P0VJM1	Os02g0518800 protein.
*Os02g0761700*	Q6Z6H2_ORYSJ	Os02g0761700 protein; it belongs to the peptidase M24A family. Methionine aminopeptidase type 1 subfamily.
*Os02g0779500*	A0A0P0VQ68	Os02g0779500 protein.
*Os02g0805800*	Q6K838_ORYSJ	Os02g0805800 protein.
*Os03g0210900*	A0A0P0VUM9	Os03g0210900 protein.
*Os03g0740900*	na	Non-protein coding transcript (cDNA clone:001-101-H08, GenBank Accession AK062364).
*Os03g0851200*	GRXS7	Monothiol glutaredoxin-S7, chloroplastic; it may only reduce GSH-thiol disulfides, but not protein disulfides; it belongs to the glutaredoxin family. CGFS subfamily.
*Os03g0852600*	Q0DLP9_ORYSJ	Os03g0852600 protein.
*Os04g0507100*	Q7X6M7_ORYSJ	cDNA clone:001-041-H09, full insert sequence.
*Os04g0661600*	C7J0Y1_ORYSJ	Molybdopterin molybdenumtransferase; it catalyzes two steps in the biosynthesis of the molybdenum cofactor. In the first step, molybdopterin is adenylated. Subsequently, molybdate is inserted into adenylated molybdopterin, and AMP is released.
*Os05g0165800*	Q60D77_ORYSJ	Os05g0165800 protein.
*Os05g0394200*	B9FPG3_ORYSJ	Os05g0394200 protein.
*Os05g0451200*	Q0DHQ3_ORYSJ	Os05g0451200 protein.
*Os05g0499600*	Q6AUW7_ORYSJ	Glycosyltransferase; it belongs to the UDP-glycosyltransferase family.
*Os06g0133800*	Q0DEU8_ORYSJ	Os06g0133800 protein.
*Os06g0364500*	Q69KT1_ORYSJ	Os06g0364500 protein.
*Os06g0618150*	A0A0P0WYR6	Os06g0618150 protein.
*Os07g0108300*	Q69UU3_ORYSJ	cDNA clone:J013118J02, full insert sequence.
*Os07g0152900*	GLO5	Peroxisomal (S)-2-hydroxy-acid oxidase GLO5; glyoxylate and dicarboxylate metabolism; photorespiratory enzyme that can exert a strong regulation over photosynthesis, possibly through a feed-back inhibition on Rubisco activase. It belongs to the FMN-dependent alpha-hydroxy acid dehydrogenase family.
*Os07g0669900*	na	Non-protein coding transcript (LOC_Os07g47360).
*Os08g0288200*	B1114E07.12	Probable adenylate kinase 5, chloroplastic; it catalyzes the reversible transfer of the terminal phosphate group between ATP and AMP. It plays an important role in cellular energy homeostasis and in adenine nucleotide metabolism.
*Os08g0483800*	A0A0P0XHG5	Os08g0483800 protein.
*Os08g0553800*	Q6Z3F9_ORYSJ	cDNA clone:001-204-F05, full insert sequence.
*Os09g0326900*	Q6K2P9_ORYSJ	cDNA clone:J013000L01, full insert sequence.
*Os09g0391501*	A0A0P0XM70	Os09g0391501 protein.
*Os11g0438500*	na	Non-protein coding transcript. (GenBank Accession AP014967)
*Os12g0502100*	B9GDA4_ORYSJ	Os12g0502100 protein.
*Os12g0638700*	A3	Plasma membrane ATPase; it belongs to the cation transport ATPase (P-type) (TC 3.A.3) family. Type IIIA subfamily.

na: not applicable. These transcripts are non-protein coding and were, therefore, not included in the construction of the STRING protein–protein interaction network.

**Table 7:** The common g:Profiler-enriched pathways that were identified in seedling/shoot via the RNA-Seq and microarray meta-analyses.

Term ID	Source	Term Name
GO:0000166	GO:MF	nucleotide binding
GO:1901363	GO:MF	heterocyclic compound binding
GO:1901265	GO:MF	nucleoside phosphate binding
GO:0043168	GO:MF	anion binding
GO:0036094	GO:MF	small molecule binding
GO:0043167	GO:MF	ion binding
GO:0003824	GO:MF	catalytic activity
KEGG:00630	KEGG	glyoxylate and dicarboxylate metabolism

## Data Availability

The original contributions presented in this study are included in the article/Supplementary Materials. Further inquiries can be directed to the corresponding author.

## Supplementary Materials

The following supporting information can be downloaded at https://www.mdpi.com/article/10.3390/ijms27073167/s1.

## Abbreviations

The following abbreviations are used in this manuscript:

ABA	Abscisic Acid
DEGs	Differentially Expressed Genes
FAOSTAT	Food and Agriculture Organization Statistics
FDR	False Discovery Rate
FPKM	Fragments Per Kilobase of Transcript Per Million Mapped Reads
NGS	Next Generation Sequencing
RAP-DB	Rice Annotation Project Database
RGAP	Rice Genome Annotation Project
ROS	Reactive Oxygen Species
RPKM	Reads Per Kilobase of Transcript Per Million Mapped Reads
TPM	Transcripts Per Million

## References

[1] Soto-Gómez D., Pérez-Rodríguez P. (2022). Sustainable Agriculture through Perennial Grains: Wheat, Rice, Maize, and Other Species. A Review. Agric. Ecosyst. Environ..

[2] FAOSTAT. https://www.fao.org/faostat/en/#data.

[3] Full Report. https://www.oecd.org/en/publications/safety-assessment-of-transgenic-organisms-in-the-environment-volume-9_e49bd2e8-en/full-report.html.

[4] Wang J., Vanga S.K., Saxena R., Orsat V., Raghavan V. (2018). Effect of Climate Change on the Yield of Cereal Crops: A Review. Climate.

[5] Saud S., Wang D., Fahad S., Alharby H.F., Bamagoos A.A., Mjrashi A., Alabdallah N.M., AlZahrani S.S., AbdElgawad H., Adnan M. (2022). Comprehensive Impacts of Climate Change on Rice Production and Adaptive Strategies in China. Front. Microbiol..

[6] Rezvi H.U.A., Tahjib-Ul-Arif M., Azim M.A., Tumpa T.A., Tipu M.M.H., Najnine F., Dawood M.F.A., Skalicky M., Brestič M. (2023). Rice and Food Security: Climate Change Implications and the Future Prospects for Nutritional Security. Food Energy Secur..

[7] Hussain S., Huang J., Huang J., Ahmad S., Nanda S., Anwar S., Shakoor A., Zhu C., Zhu L., Cao X., Fahad S., Hasanuzzaman M., Alam M., Ullah H., Saeed M., Ali Khan I., Adnan M. (2020). Rice Production Under Climate Change: Adaptations and Mitigating Strategies. Environment, Climate, Plant and Vegetation Growth.

[8] Khan M.I.R., Palakolanu S.R., Chopra P., Rajurkar A.B., Gupta R., Iqbal N., Maheshwari C. (2021). Improving Drought Tolerance in Rice: Ensuring Food Security through Multi-Dimensional Approaches. Physiol. Plant..

[9] Kumar A., Sengar R.S., Pathak R.K., Singh A.K. (2023). Integrated Approaches to Develop Drought-Tolerant Rice: Demand of Era for Global Food Security. J. Plant Growth Regul..

[10] Panda D., Mishra S.S., Behera P.K. (2021). Drought Tolerance in Rice: Focus on Recent Mechanisms and Approaches. Rice Sci..

[11] Oladosu Y., Rafii M.Y., Samuel C., Fatai A., Magaji U., Kareem I., Kamarudin Z.S., Muhammad I., Kolapo K. (2019). Drought Resistance in Rice from Conventional to Molecular Breeding: A Review. Int. J. Mol. Sci..

[12] Martin L., Fei Z., Giovannoni J., Rose J.K.C. (2013). Catalyzing Plant Science Research with RNA-Seq. Front. Plant Sci..

[13] Govindaraj M., Vetriventhan M., Srinivasan M. (2015). Importance of Genetic Diversity Assessment in Crop Plants and Its Recent Advances: An Overview of Its Analytical Perspectives. Genet. Res. Int..

[14] Feuillet C., Leach J.E., Rogers J., Schnable P.S., Eversole K. (2011). Crop Genome Sequencing: Lessons and Rationales. Trends Plant Sci..

[15] Rensink W.A., Buell C.R. (2005). Microarray Expression Profiling Resources for Plant Genomics. Trends Plant Sci..

[16] Zhou X., Bai X., Xing Y. (2018). A Rice Genetic Improvement Boom by Next-Generation Sequencing. Curr. Issues Mol. Biol..

[17] Swamy B.P.M., Kumar A. (2013). Genomics-Based Precision Breeding Approaches to Improve Drought Tolerance in Rice. Biotechnol. Adv..

[18] Tamura K., Bono H. (2022). Meta-Analysis of RNA Sequencing Data of Arabidopsis and Rice under Hypoxia. Life.

[19] Borenstein M., Hedges L.V., Higgins J.P.T., Rothstein H.R. (2021). Introduction to Meta-Analysis.

[20] Toro-Domínguez D., Villatoro-García J.A., Martorell-Marugán J., Román-Montoya Y., Alarcón-Riquelme M.E., Carmona-Sáez P. (2021). A Survey of Gene Expression Meta-Analysis: Methods and Applications. Brief. Bioinform..

[21] Suravajhala P.N., Bizzaro J.W. (2025). Next-Generation Sequencing: Standard Operating Procedures and Applications.

[22] Tseng G.C., Ghosh D., Feingold E. (2012). Comprehensive Literature Review and Statistical Considerations for Microarray Meta-Analysis. Nucleic Acids Res..

[23] Hong F., Breitling R. (2008). A Comparison of Meta-Analysis Methods for Detecting Differentially Expressed Genes in Microarray Experiments. Bioinformatics.

[24] Chen K., Du K., Shi Y., Yin L., Shen W.-H., Yu Y., Liu B., Dong A. (2021). H3K36 Methyltransferase SDG708 Enhances Drought Tolerance by Promoting Abscisic Acid Biosynthesis in Rice. New Phytol..

[25] Sevanthi A.M., Sinha S.K., V S., Rani M., Saini M.R., Kumari S., Kaushik M., Prakash C., K V., Singh G.P. (2021). Integration of Dual Stress Transcriptomes and Major QTLs from a Pair of Genotypes Contrasting for Drought and Chronic Nitrogen Starvation Identifies Key Stress Responsive Genes in Rice. Rice.

[26] Ma S., Tang N., Li X., Xie Y., Xiang D., Fu J., Shen J., Yang J., Tu H., Li X. (2019). Reversible Histone H2B Monoubiquitination Fine-Tunes Abscisic Acid Signaling and Drought Response in Rice. Mol. Plant.

[27] Li X., Chang Y., Ma S., Shen J., Hu H., Xiong L. (2019). Genome-Wide Identification of SNAC1-Targeted Genes Involved in Drought Response in Rice. Front. Plant Sci..

[28] Fu J., Wu H., Ma S., Xiang D., Liu R., Xiong L. (2017). OsJAZ1 Attenuates Drought Resistance by Regulating JA and ABA Signaling in Rice. Front. Plant Sci..

[29] Yang J., Chang Y., Qin Y., Chen D., Zhu T., Peng K., Wang H., Tang N., Li X., Wang Y. (2020). A Lamin-like Protein OsNMCP1 Regulates Drought Resistance and Root Growth through Chromatin Accessibility Modulation by Interacting with a Chromatin Remodeller OsSWI3C in Rice. New Phytol..

[30] Ogawa D., Suzuki Y., Yokoo T., Katoh E., Teruya M., Muramatsu M., Ma J.F., Yoshida Y., Isaji S., Ogo Y. (2021). Acetic-Acid-Induced Jasmonate Signaling in Root Enhances Drought Avoidance in Rice. Sci. Rep..

[31] Bhattacharjee A., Sharma R., Jain M. (2017). Over-Expression of OsHOX24 Confers Enhanced Susceptibility to Abiotic Stresses in Transgenic Rice via Modulating Stress-Responsive Gene Expression. Front. Plant Sci..

[32] Borah P., Sharma E., Kaur A., Chandel G., Mohapatra T., Kapoor S., Khurana J.P. (2017). Analysis of Drought-Responsive Signalling Network in Two Contrasting Rice Cultivars Using Transcriptome-Based Approach. Sci. Rep..

[33] Singh A., Giri J., Kapoor S., Tyagi A.K., Pandey G.K. (2010). Protein Phosphatase Complement in Rice: Genome-Wide Identification and Transcriptional Analysis under Abiotic Stress Conditions and Reproductive Development. BMC Genom..

[34] Muthurajan R., Rahman H., Manoharan M., Ramanathan V., Nallathambi J. (2018). Drought Responsive Transcriptome Profiling in Roots of Contrasting Rice Genotypes. Indian J. Plant Physiol..

[35] Dixit S., Kumar Biswal A., Min A., Henry A., Oane R.H., Raorane M.L., Longkumer T., Pabuayon I.M., Mutte S.K., Vardarajan A.R. (2015). Action of Multiple Intra-QTL Genes Concerted around a Co-Localized Transcription Factor Underpins a Large Effect QTL. Sci. Rep..

[36] Conlon E.M., Song J.J., Liu A. (2007). Bayesian Meta-Analysis Models for Microarray Data: A Comparative Study. BMC Bioinform..

[37] Choi J.K., Yu U., Kim S., Yoo O.J. (2003). Combining Multiple Microarray Studies and Modeling Interstudy Variation. Bioinformatics.

[38] Vennou K.E., Kontou P.I., Braliou G.G., Bagos P.G. (2020). Meta-Analysis of Gene Expression Profiles in Preeclampsia. Pregnancy Hypertens..

[39] Raudvere U., Kolberg L., Kuzmin I., Arak T., Adler P., Peterson H., Vilo J. (2019). G:Profiler: A Web Server for Functional Enrichment Analysis and Conversions of Gene Lists (2019 Update). Nucleic Acids Res..

[40] Hassan M.A., Dahu N., Hongning T., Qian Z., Yueming Y., Yiru L., Shimei W. (2023). Drought Stress in Rice: Morpho-Physiological and Molecular Responses and Marker-Assisted Breeding. Front. Plant Sci..

[41] Bhandari U., Gajurel A., Khadka B., Thapa I., Chand I., Bhatta D., Poudel A., Pandey M., Shrestha S., Shrestha J. (2023). Morpho-Physiological and Biochemical Response of Rice (*Oryza sativa* L.) to Drought Stress: A Review. Heliyon.

[42] Pandey V., Shukla A. (2015). Acclimation and Tolerance Strategies of Rice under Drought Stress. Rice Sci..

[43] Nakashima K., Yamaguchi-Shinozaki K., Shinozaki K. (2014). The Transcriptional Regulatory Network in the Drought Response and Its Crosstalk in Abiotic Stress Responses Including Drought, Cold, and Heat. Front. Plant Sci..

[44] Koricheva J., Gurevitch J., Mengersen K. (2013). Handbook of Meta-Analysis in Ecology and Evolution.

[45] Dorn A., Puchta H. (2019). DNA Helicases as Safekeepers of Genome Stability in Plants. Genes.

[46] Byrne M.E. (2009). A Role for the Ribosome in Development. Trends Plant Sci..

[47] Schmid L.-M., Manavski N., Chi W., Meurer J. (2024). Chloroplast Ribosome Biogenesis Factors. Plant Cell Physiol..

[48] Atkin O.K., Macherel D. (2009). The Crucial Role of Plant Mitochondria in Orchestrating Drought Tolerance. Ann. Bot..

[49] Muhammad I., Shalmani A., Ali M., Yang Q.-H., Ahmad H., Li F.B. (2021). Mechanisms Regulating the Dynamics of Photosynthesis Under Abiotic Stresses. Front. Plant Sci..

[50] Suhorukova A.V., Sobolev D.S., Milovskaya I.G., Fadeev V.S., Goldenkova-Pavlova I.V., Tyurin A.A. (2023). A Molecular Orchestration of Plant Translation under Abiotic Stress. Cells.

[51] Allan W.L., Clark S.M., Hoover G.J., Shelp B.J. (2009). Role of Plant Glyoxylate Reductases during Stress: A Hypothesis. Biochem. J..

[52] Jiang X., Walker B.J., He S.Y., Hu J. (2023). The Role of Photorespiration in Plant Immunity. Front. Plant Sci..

[53] Nie P., Li X., Liu Y., Zhang Q. (2011). Expression Analysis of Nuclear W2-Containing Homologs of Eukaryotic Initiation Factors in Rice. Biologia.

[54] Aki T., Yanagisawa S. (2009). Application of Rice Nuclear Proteome Analysis to the Identification of Evolutionarily Conserved and Glucose-Responsive Nuclear Proteins. J. Proteome Res..

[55] Guo Z., Cai L., Liu C., Huang C., Chen Z., Pan G., Guo T. (2020). Global Analysis of Differentially Expressed Genes between Two Japonica Rice Varieties Induced by Low Temperature during the Booting Stage by RNA-Seq. R. Soc. Open Sci..

[56] Gan P., Luo X., Wei H., Hu Y., Li R., Luo J. (2023). Identification of Hub Genes That Variate the qCSS12-Mediated Cold Tolerance between Indica and Japonica Rice Using WGCNA. Plant Cell Rep..

[57] Yang D.-L., Yang Y., He Z. (2013). Roles of Plant Hormones and Their Interplay in Rice Immunity. Mol. Plant.

[58] Verma V., Ravindran P., Kumar P.P. (2016). Plant Hormone-Mediated Regulation of Stress Responses. BMC Plant Biol..

[59] Sharma R., Vleesschauwer D.D., Sharma M.K., Ronald P.C. (2013). Recent Advances in Dissecting Stress-Regulatory Crosstalk in Rice. Mol. Plant.

[60] Aluko O.O., Li C., Wang Q., Liu H. (2021). Sucrose Utilization for Improved Crop Yields: A Review Article. Int. J. Mol. Sci..

[61] Thomas A., Beena R. (2024). Sucrose Metabolism in Plants under Drought Stress Condition: A Review. Indian J. Agric. Res..

[62] Delatte T., Umhang M., Trevisan M., Eicke S., Thorneycroft D., Smith S.M., Zeeman S.C. (2006). Evidence for Distinct Mechanisms of Starch Granule Breakdown in Plants*. J. Biol. Chem..

[63] Patindol J.A., Siebenmorgen T.J., Wang Y.-J. (2015). Impact of Environmental Factors on Rice Starch Structure: A Review. Starch-Stärke.

[64] Mansoor S., Khan T., Farooq I., Shah L.R., Sharma V., Sonne C., Rinklebe J., Ahmad P. (2022). Drought and Global Hunger: Biotechnological Interventions in Sustainability and Management. Planta.

[65] Raplee I.D., Borkar S.A., Yin L., Venturi G.M., Shen J., Chang K.-F., Nepal U., Sleasman J.W., Goodenow M.M. (2025). The Role of Microarray in Modern Sequencing: Statistical Approach Matters in a Comparison Between Microarray and RNA-Seq. BioTech.

[66] Kim W.-J., Choi B.R., Noh J.J., Lee Y.-Y., Kim T.-J., Lee J.-W., Kim B.-G., Choi C.H. (2024). Comparison of RNA-Seq and Microarray in the Prediction of Protein Expression and Survival Prediction. Front. Genet..

[67] Khadka V.S., Vaughn K., Xie J., Swaminathan P., Ma Q., Cramer G.R., Fennell A.Y. (2019). Transcriptomic Response Is More Sensitive to Water Deficit in Shoots than Roots of *Vitis riparia* (Michx.). BMC Plant Biol..

[68] Turowski V.R., Aknin C., Maliandi M.V., Buchensky C., Leaden L., Peralta D.A., Busi M.V., Araya A., Gomez-Casati D.F. (2015). Frataxin Is Localized to Both the Chloroplast and Mitochondrion and Is Involved in Chloroplast Fe-S Protein Function in Arabidopsis. PLoS ONE.

[69] Özmen C.Y., Baydu F.Y., Ergül A., Özmen C.Y., Baydu F.Y., Ergül A. (2025). Comparative Analysis of Cabernet Sauvignon (*Vitis vinifera* L.) and Kober 5BB (*V. berlandieri* × *V. riparia*) Root Transcriptomes Reveals Multiple Processes Associated with Drought Tolerance in Grapevines. Horticulturae.

[70] Hao Z., Ma S., Liang L., Feng T., Xiong M., Lian S., Zhu J., Chen Y., Meng L., Li M. (2022). Candidate Genes and Pathways in Rice Co-Responding to Drought and Salt Identified by gcHap Network. Int. J. Mol. Sci..

[71] Sircar S., Parekh N. (2019). Meta-Analysis of Drought-Tolerant Genotypes in Oryza Sativa: A Network-Based Approach. PLoS ONE.

[72] Selamat N., Nadarajah K.K., Selamat N., Nadarajah K.K. (2021). Meta-Analysis of Quantitative Traits Loci (QTL) Identified in Drought Response in Rice (*Oryza sativa* L.). Plants.

[73] Nazarov P.V., Muller A., Kaoma T., Nicot N., Maximo C., Birembaut P., Tran N.L., Dittmar G., Vallar L. (2017). RNA Sequencing and Transcriptome Arrays Analyses Show Opposing Results for Alternative Splicing in Patient Derived Samples. BMC Genom..

[74] Seyednasrollah F., Laiho A., Elo L.L. (2015). Comparison of Software Packages for Detecting Differential Expression in RNA-Seq Studies. Brief. Bioinform..

[75] Zhang W., Yu Y., Hertwig F., Thierry-Mieg J., Zhang W., Thierry-Mieg D., Wang J., Furlanello C., Devanarayan V., Cheng J. (2015). Comparison of RNA-Seq and Microarray-Based Models for Clinical Endpoint Prediction. Genome Biol..

[76] Corchete L.A., Rojas E.A., Alonso-López D., De Las Rivas J., Gutiérrez N.C., Burguillo F.J. (2020). Systematic Comparison and Assessment of RNA-Seq Procedures for Gene Expression Quantitative Analysis. Sci. Rep..

[77] Sîrbu A., Kerr G., Crane M., Ruskin H.J. (2012). RNA-Seq vs Dual-and Single-Channel Microarray Data: Sensitivity Analysis for Differential Expression and Clustering. PLoS ONE.

[78] Deyneko I.V., Mustafaev O.N., Tyurin A.A., Zhukova K.V., Varzari A., Goldenkova-Pavlova I.V. (2022). Modeling and Cleaning RNA-Seq Data Significantly Improve Detection of Differentially Expressed Genes. BMC Bioinform..

[79] Jose J., Ghantasala S., Roy Choudhury S. (2020). Arabidopsis Transmembrane Receptor-Like Kinases (RLKs): A Bridge between Extracellular Signal and Intracellular Regulatory Machinery. Int. J. Mol. Sci..

[80] Zhang Z., Li X., Cui L., Meng S., Ye N., Peng X. (2017). Catalytic and Functional Aspects of Different Isozymes of Glycolate Oxidase in Rice. BMC Plant Biol..

[81] Li M., Guo P., Nan N., Ma A., Liu W., Wang T.-J., Yun D.-J., Xu Z.-Y. (2023). Plasma Membrane-Localized H+-ATPase OsAHA3 Functions in Saline–Alkaline Stress Tolerance in Rice. Plant Cell Rep..

[82] Zhai R., Ye S., Ye J., Wu M., Zhu G., Yu F., Wang X., Feng Y., Zhang X. (2023). Glutaredoxin in Rice Growth, Development, and Stress Resistance: Mechanisms and Research Advances. Int. J. Mol. Sci..

[83] Lu X., Zhang D., Zhang Y., Liu X., Wang S., Liu X. (2023). The Molybdenum Cofactor Biosynthesis Gene, OsCNX1, Is Essential for Seedling Development and Seed Germination in Rice. Mol. Breed..

[84] Yoo Y.-H., Jiang X., Jung K.-H. (2020). An Abiotic Stress Responsive U-Box E3 Ubiquitin Ligase Is Involved in OsGI-Mediating Diurnal Rhythm Regulating Mechanism. Plants.

[85] Desaki Y., Takahashi S., Sato K., Maeda K., Matsui S., Yoshimi I., Miura T., Jumonji J.-I., Takeda J., Yashima K. (2019). PUB4, a CERK1-Interacting Ubiquitin Ligase, Positively Regulates MAMP-Triggered Immunity in Arabidopsis. Plant Cell Physiol..

[86] Derakhshani B., Lee C., Shin D., Jung K.-H. (2024). Identification of Core Drought-Responsive Genes for Developing Drought-Tolerant Rice Varieties through Meta-Analysis of RNA-Seq Data. Plant Biotechnol. Rep..

[87] Sirohi P., Yadav B.S., Afzal S., Mani A., Singh N.K. (2020). Identification of Drought Stress-Responsive Genes in Rice (*Oryza sativa*) by Meta-Analysis of Microarray Data. J. Genet..

[88] Singh B.K., Venkadesan S., Ramkumar M.K., Shanmugavadivel P.S., Dutta B., Prakash C., Pal M., Solanke A.U., Rai A., Singh N.K. (2023). Meta-Analysis of Microarray Data and Their Utility in Dissecting the Mapped QTLs for Heat Acclimation in Rice. Plants.

[89] Ma T., Liang F., Oesterreich S., Tseng G.C. (2017). A Joint Bayesian Model for Integrating Microarray and RNA Sequencing Transcriptomic Data. J. Comput. Biol..

[90] Naithani S., Gupta P., Preece J., Garg P., Fraser V., Padgitt-Cobb L.K., Martin M., Vining K., Jaiswal P. (2019). Involving Community in Genes and Pathway Curation. Database.

[91] Page M.J., McKenzie J.E., Bossuyt P.M., Boutron I., Hoffmann T.C., Mulrow C.D., Shamseer L., Tetzlaff J.M., Akl E.A., Brennan S.E. (2021). The PRISMA 2020 Statement: An Updated Guideline for Reporting Systematic Reviews. BMJ.

[92] Barrett T., Wilhite S.E., Ledoux P., Evangelista C., Kim I.F., Tomashevsky M., Marshall K.A., Phillippy K.H., Sherman P.M., Holko M. (2013). NCBI GEO: Archive for Functional Genomics Data Sets—Update. Nucleic Acids Res..

[93] Kawahara Y., de la Bastide M., Hamilton J.P., Kanamori H., McCombie W.R., Ouyang S., Schwartz D.C., Tanaka T., Wu J., Zhou S. (2013). Improvement of the Oryza Sativa Nipponbare Reference Genome Using next Generation Sequence and Optical Map Data. Rice.

[94] Hamilton J.P., Li C., Buell C.R. (2025). The Rice Genome Annotation Project: An Updated Database for Mining the Rice Genome. Nucleic Acids Res..

[95] Sakai H., Lee S.S., Tanaka T., Numa H., Kim J., Kawahara Y., Wakimoto H., Yang C., Iwamoto M., Abe T. (2013). Rice Annotation Project Database (RAP-DB): An Integrative and Interactive Database for Rice Genomics. Plant Cell Physiol..

[96] Li B., Dewey C.N. (2011). RSEM: Accurate Transcript Quantification from RNA-Seq Data with or without a Reference Genome. BMC Bioinform..

[97] Mortazavi A., Williams B.A., McCue K., Schaeffer L., Wold B. (2008). Mapping and Quantifying Mammalian Transcriptomes by RNA-Seq. Nat. Methods.

[98] Trapnell C., Williams B.A., Pertea G., Mortazavi A., Kwan G., van Baren M.J., Salzberg S.L., Wold B.J., Pachter L. (2010). Transcript Assembly and Quantification by RNA-Seq Reveals Unannotated Transcripts and Isoform Switching during Cell Differentiation. Nat. Biotechnol..

[99] Wagner G.P., Kin K., Lynch V.J. (2012). Measurement of mRNA Abundance Using RNA-Seq Data: RPKM Measure Is Inconsistent among Samples. Theory Biosci..

[100] Ramasamy A., Mondry A., Holmes C.C., Altman D.G. (2008). Key Issues in Conducting a Meta-Analysis of Gene Expression Microarray Datasets. PLoS Med..

[101] Campain A., Yang Y.H. (2010). Comparison Study of Microarray Meta-Analysis Methods. BMC Bioinform..

[102] Cohen J. (2013). Statistical Power Analysis for the Behavioral Sciences.

[103] Hedges L.V. (1981). Distribution Theory for Glass’s Estimator of Effect Size and Related Estimators. J. Educ. Stat..

[104] Kontou P.I., Pavlopoulou A., Bagos P.G., Evangelou E. (2018). Methods of Analysis and Meta-Analysis for Identifying Differentially Expressed Genes. Genetic Epidemiology: Methods and Protocols.

[105] Hedges L.V., Olkin I. (2014). Statistical Methods for Meta-Analysis.

[106] Cochran W.G. (1954). The Combination of Estimates from Different Experiments. Biometrics.

[107] Higgins J.P.T., Thompson S.G. (2002). Quantifying Heterogeneity in a Meta-Analysis. Stat. Med..

[108] DerSimonian R., Laird N. (1986). Meta-Analysis in Clinical Trials. Control. Clin. Trials.

[109] Dudoit S., Yang Y.H., Callow M.J., Speed T.P. (2002). Statistical Methods for Identifying Differentially Expressed Genes in Replicated cDNA Microarray Experiments. Stat. Sin..

[110] Šidák Z. (1967). Rectangular Confidence Regions for the Means of Multivariate Normal Distributions. J. Am. Stat. Assoc..

[111] Holland B.S., Copenhaver M.D. (1988). Improved Bonferroni-Type Multiple Testing Procedures. Psychol. Bull..

[112] Holm S. (1979). A Simple Sequentially Rejective Multiple Test Procedure. Scand. J. Stat..

[113] Benjamini Y., Hochberg Y. (1995). Controlling the False Discovery Rate: A Practical and Powerful Approach to Multiple Testing. J. R. Stat. Soc. Ser. B Methodol..

[114] Tamposis I.A., Manios G.A., Charitou T., Vennou K.E., Kontou P.I., Bagos P.G. (2022). MAGE: An Open-Source Tool for Meta-Analysis of Gene Expression Studies. Biology.

[115] Szklarczyk D., Nastou K., Koutrouli M., Kirsch R., Mehryary F., Hachilif R., Hu D., Peluso M.E., Huang Q., Fang T. (2025). The STRING Database in 2025: Protein Networks with Directionality of Regulation. Nucleic Acids Res..

[116] Mi H., Ebert D., Muruganujan A., Mills C., Albou L.-P., Mushayamaha T., Thomas P.D. (2020). PANTHER Version 16: A Revised Family Classification, Tree-Based Classification Tool, Enhancer Regions and Extensive API. Nucleic Acids Res..

[117] Thomas P.D., Ebert D., Muruganujan A., Mushayahama T., Albou L.-P., Mi H. (2022). PANTHER: Making Genome-Scale Phylogenetics Accessible to All. Protein Sci..

[118] Van Dongen S. (2008). Graph Clustering via a Discrete Uncoupling Process. SIAM J. Matrix Anal. Appl..

[119] Srihari S., Ning K., Leong H.W. (2010). MCL-CAw: A Refinement of MCL for Detecting Yeast Complexes from Weighted PPI Networks by Incorporating Core-Attachment Structure. BMC Bioinform..

[120] Letunic I., Khedkar S., Bork P. (2021). SMART: Recent Updates, New Developments and Status in 2020. Nucleic Acids Res..

[121] The UniProt Consortium (2025). UniProt: The Universal Protein Knowledgebase in 2025. Nucleic Acids Res..

