# Supplementary material for: Transcriptomic Landscape and Regulatory Pathways of Drought Response in Rice (Oryza sativa L.): A Meta-Analysis of Microarray and RNA-Seq Data
Source: Int J Mol Sci. 2026 Mar 31;27(7):3167. doi: 10.3390/ijms27073167 (PMC13074122; doi:10.3390/ijms27073167)
Supplement: Supplementary file 1 [file ijms-27-03167-s001.zip › Supplementary Figure S1.pptx]

## Slide 1
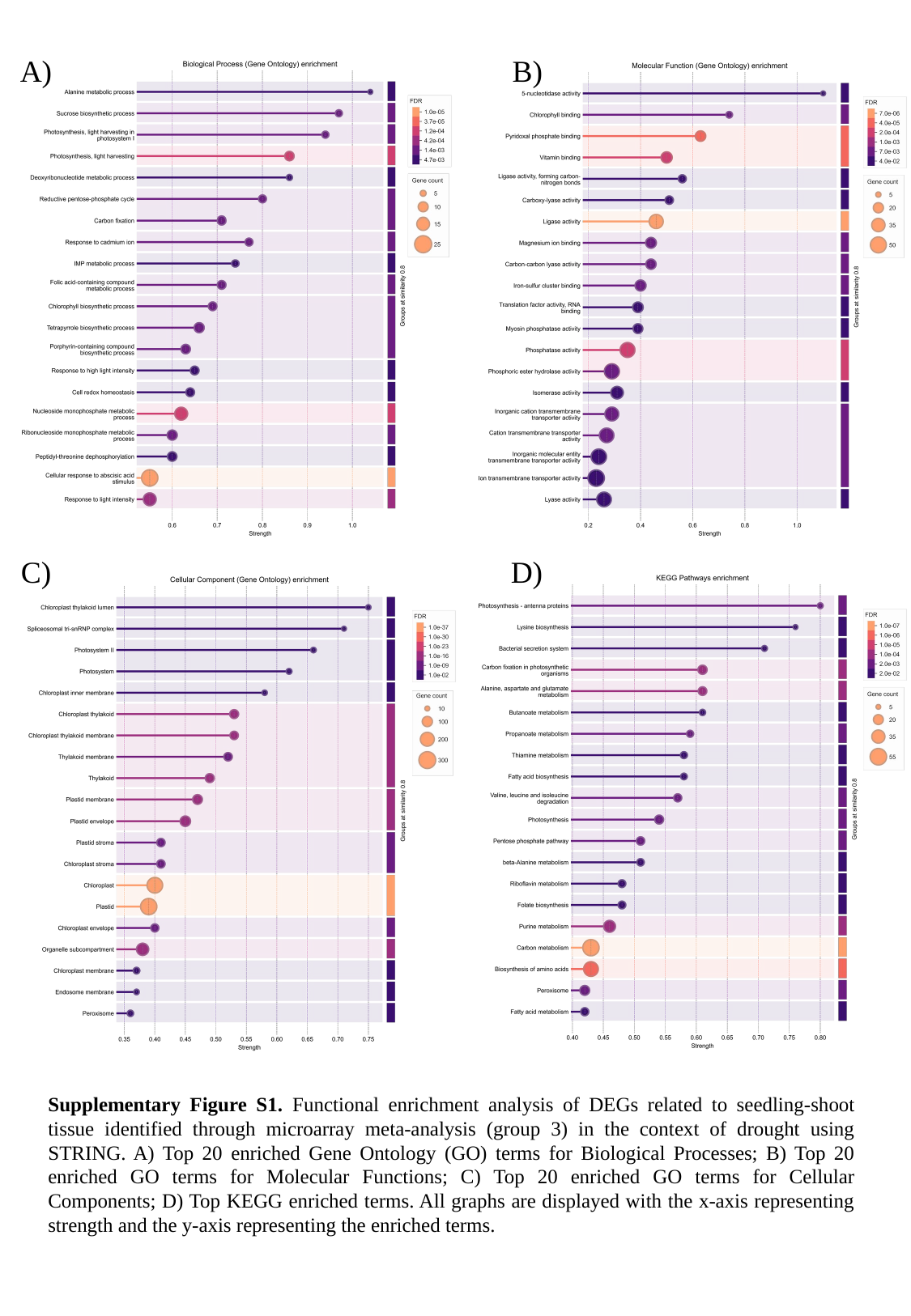

A)
B)
C)
D)
Supplementary Figure S1. Functional enrichment analysis of DEGs related to seedling-shoot tissue identified through microarray meta-analysis (group 3) in the context of drought using STRING. A) Top 20 enriched Gene Ontology (GO) terms for Biological Processes; B) Top 20 enriched GO terms for Molecular Functions; C) Top 20 enriched GO terms for Cellular Components; D) Top KEGG enriched terms. All graphs are displayed with the x-axis representing strength and the y-axis representing the enriched terms.
