# Supplementary material for: Transcriptomic Landscape and Regulatory Pathways of Drought Response in Rice (Oryza sativa L.): A Meta-Analysis of Microarray and RNA-Seq Data
Source: Int J Mol Sci. 2026 Mar 31;27(7):3167. doi: 10.3390/ijms27073167 (PMC13074122; doi:10.3390/ijms27073167)
Supplement: Supplementary file 1 [file ijms-27-03167-s001.zip › Supplementary Figure S2.pptx]

## Slide 1
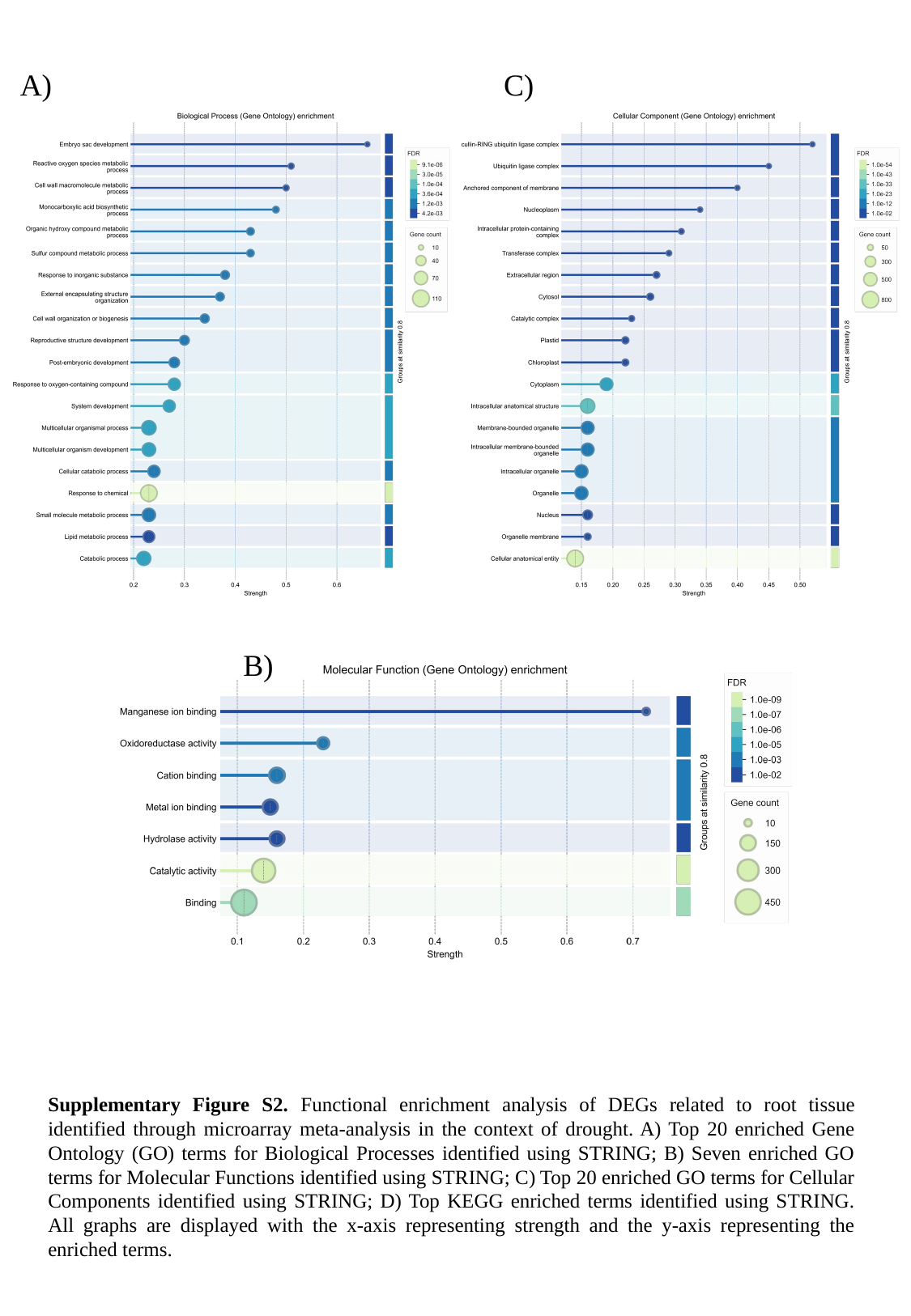

A)
C)
B)
Supplementary Figure S2. Functional enrichment analysis of DEGs related to root tissue identified through microarray meta-analysis in the context of drought. A) Top 20 enriched Gene Ontology (GO) terms for Biological Processes identified using STRING; B) Seven enriched GO terms for Molecular Functions identified using STRING; C) Top 20 enriched GO terms for Cellular Components identified using STRING; D) Top KEGG enriched terms identified using STRING. All graphs are displayed with the x-axis representing strength and the y-axis representing the enriched terms.
