# Supplementary material for: Transcriptomic Landscape and Regulatory Pathways of Drought Response in Rice (Oryza sativa L.): A Meta-Analysis of Microarray and RNA-Seq Data
Source: Int J Mol Sci. 2026 Mar 31;27(7):3167. doi: 10.3390/ijms27073167 (PMC13074122; doi:10.3390/ijms27073167)
Supplement: Supplementary file 1 [file ijms-27-03167-s001.zip › Supplementary Figure S3.pptx]

## Slide 1
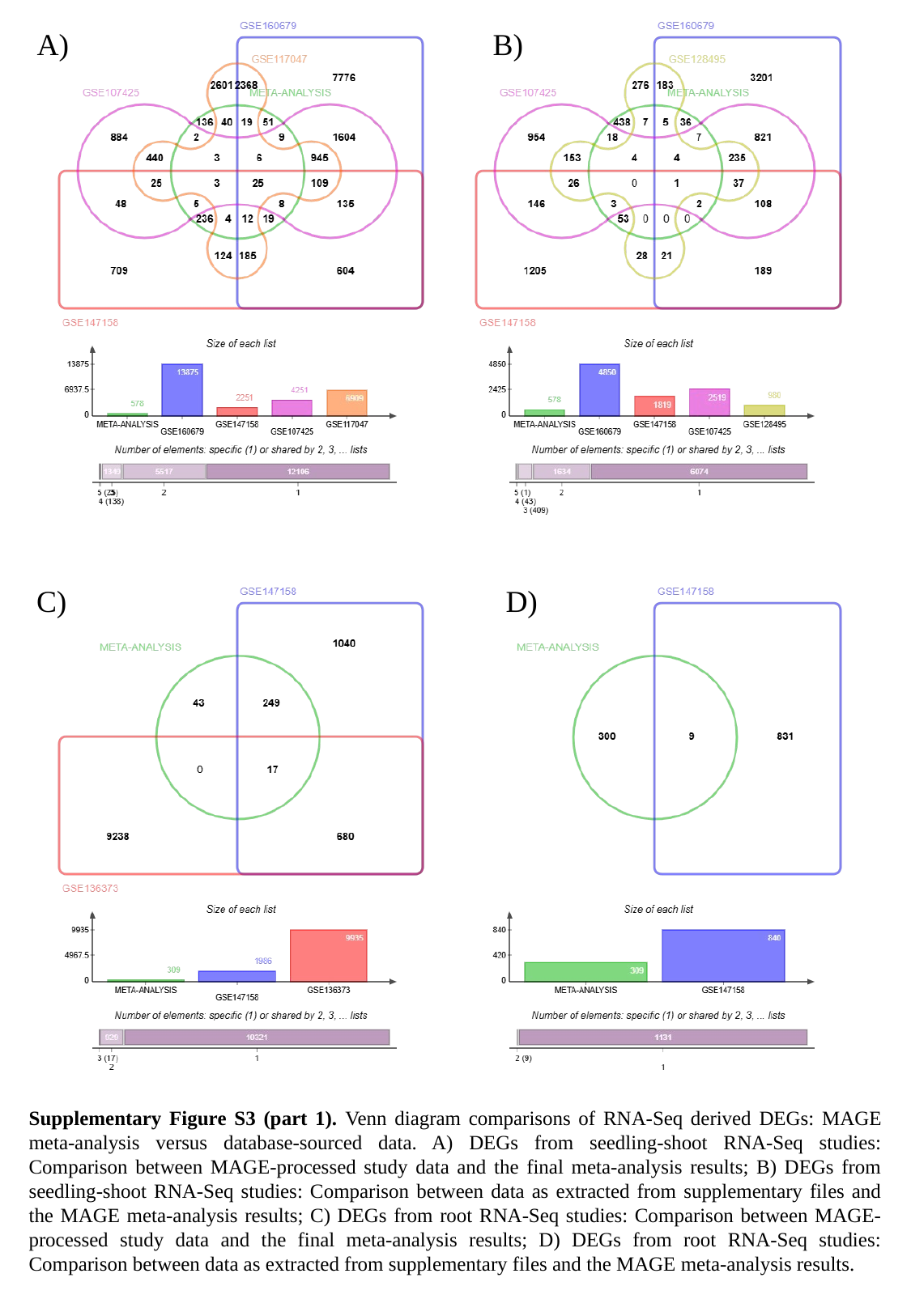

A)
B)
D)
C)
Supplementary Figure S3 (part 1). Venn diagram comparisons of RNA-Seq derived DEGs: MAGE meta-analysis versus database-sourced data. A) DEGs from seedling-shoot RNA-Seq studies: Comparison between MAGE-processed study data and the final meta-analysis results; B) DEGs from seedling-shoot RNA-Seq studies: Comparison between data as extracted from supplementary files and the MAGE meta-analysis results; C) DEGs from root RNA-Seq studies: Comparison between MAGE-processed study data and the final meta-analysis results; D) DEGs from root RNA-Seq studies: Comparison between data as extracted from supplementary files and the MAGE meta-analysis results.

## Slide 2
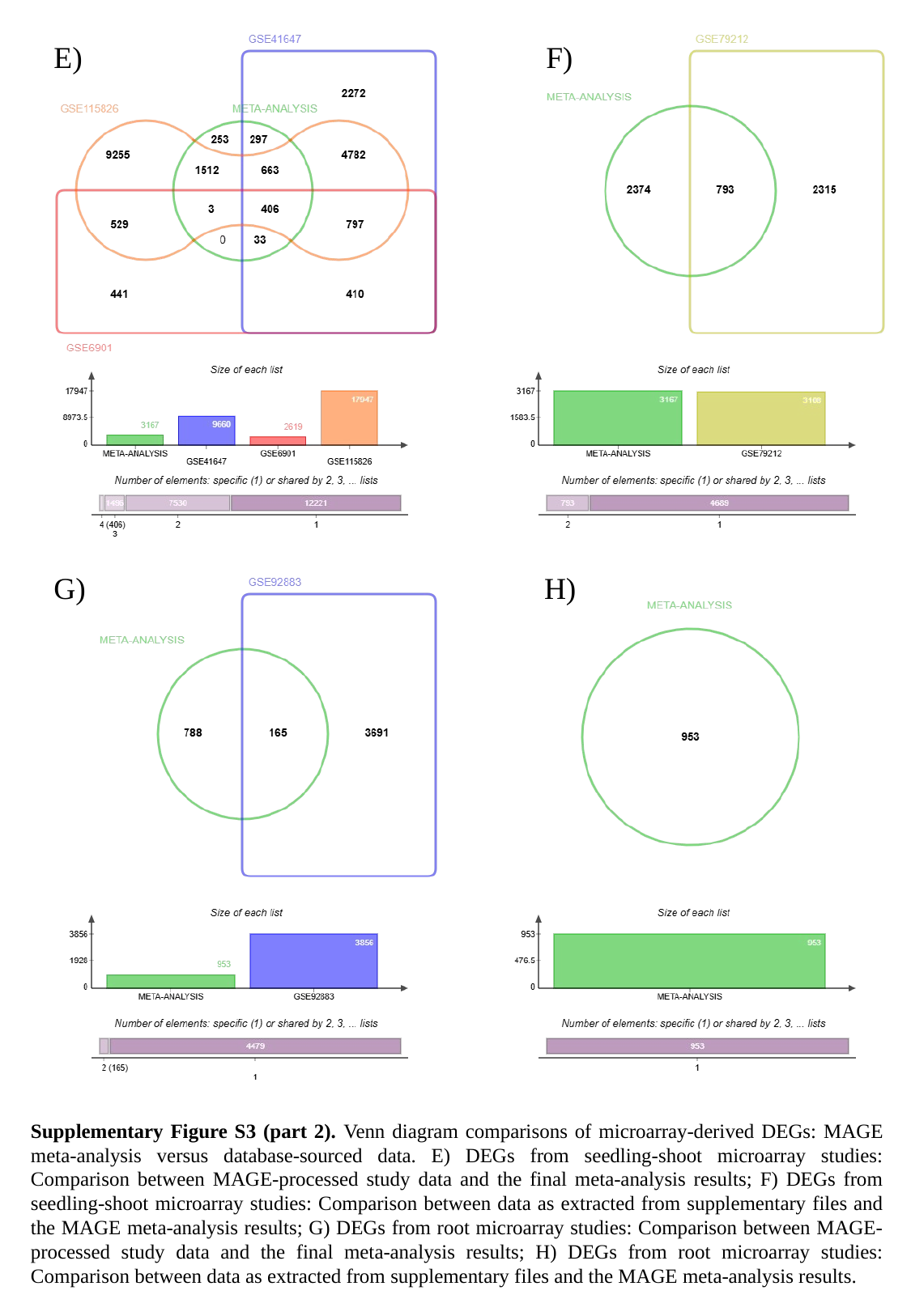

E)
F)
G)
H)
Supplementary Figure S3 (part 2). Venn diagram comparisons of microarray-derived DEGs: MAGE meta-analysis versus database-sourced data. E) DEGs from seedling-shoot microarray studies: Comparison between MAGE-processed study data and the final meta-analysis results; F) DEGs from seedling-shoot microarray studies: Comparison between data as extracted from supplementary files and the MAGE meta-analysis results; G) DEGs from root microarray studies: Comparison between MAGE-processed study data and the final meta-analysis results; H) DEGs from root microarray studies: Comparison between data as extracted from supplementary files and the MAGE meta-analysis results.
